# Supplementary material for: Community health workers’ efforts to build health system trust in marginalised communities: a qualitative study from South Africa
Source: BMJ Open. 2021 May 19;11(5):e044065. doi: 10.1136/bmjopen-2020-044065 (PMC8137175; doi:10.1136/bmjopen-2020-044065)
Supplement: Supplementary data [file bmjopen-2020-044065supp001.pdf]

V0.95; 12 September 2016

Interview Guides for the Bathlokomedi project

<u>Annex A-G</u>

Annex A: Information Sheet for interviewing the facility manager of the 'mother' clinic.

Annex B: Information Sheet for interviewing Nurse team leader of the CHW team and Field version Nurse team leader of CHW interviews

Annex C: Information Sheet for observing community health workers

Annex D: Information Sheet for observing nurse leader of community health worker team

Annex E: Information sheet for group interview with community health workers

Annex G: Information Sheet for interviewing referred householder

Annex A: Information Sheet for interviewing the facility manager of the 'mother' clinic.

Formal Title: Implementing comprehensive, integrated, community-based health care for under-served, vulnerable communities in South Africa: A practical, evidence-informed model.

Hello, and thank you for your time today.

My name is ________. I work for the Centre for Health Policy at the University of the Witwatersrand. I am a researcher in the Batlhokomedi project, and I would like to interview you today as part of this project.

<u>What is this research about?</u>
We are conducting this research to understand how community health worker / WBOT programmes operate. We know that there are some innovative WBOT programmes in Sedibeng and we are keen to learn more about your programmes here. I believe you are the manager of this clinic that has some WBOT teams. So we would like to hear about your experiences of having WBOTs associated with your clinic, both the successes and challenges.

The interview should take about 40 mins.

<u>Voluntary Participation</u>
It is up to you to decide whether you want participate in this study. If you agree to help with this research, and later change your mind, you are free to end the interview at any time, without any consequences.

<u>Risks</u>
There are no risks associated with talking to me today.

Anstey Watkins J, *et al. BMJ Open* 2021; 11:e044065. doi: 10.1136/bmjopen-2020-044065

V0.95; 12 September 2016

Confidentiality
No one other than my team members will be allowed to see the record of the interview discussion. We will not use your name in any reports of this work. We will use a code instead of your name e.g. A or B.

My notes and audio recordings will be kept on a secure university computer server, and participant codes will be kept in a locked filing cabinet under the care of the project site manager, and will be destroyed once our research is complete.

Approval for and benefits of this work
The study has been approved by the ethics committee of University of the Witwatersrand, and Gauteng Province. We hope the study will contribute new ideas and insights regarding the provision of community health worker services.

If you agree to be interviewed, we would also like your permission to record the interview as it really helps later in the office.

What if I have any questions?
You are free to ask me any question about this research. If you have any further questions about the study, you are free to contact Julia De Kadt on Julia.DeKadt2@wits.ac.za, or 011-717-3434 or 074 336 9411.
If you are concerned about anything to do with the study in general, or wish to make a complaint, you can contact the chair of the ethics committee at the University of the Witwatersrand, who is Professor Peter Cleaton-Jones at telephone number: (011) 717-2301.

Tool A: Interview managers of the 'mother' clinic to which the CHW team

Introduction
Thank you again for agreeing to be interviewed. Are you happy to start?

First, I want to ask you some questions about yourself. Please tell me a bit about yourself?

Probes:
How many years have you worked as a nurse?
What is your professional title? (eg. Enrolled nurse, Professional nurse, other specialized nurse)
How many years have you worked at this facility?
How many years in the current position?
Were you born in this district?

Perhaps we could talk now about the relationship between the clinic and the CHW team. Please can you describe the relationship between the 'mother' clinic and the CHW team?

Probes:
How long has the CHW team been working from / linked to this clinic?
What role does the clinic play in the recruitment of CHWs?
What role the 'mother' clinic has in supporting the CHW team?
What happens when patients are referred here by CHW? Who do they see?
How does the data reporting system work?
What else could the CHWs do to better support the clinic?

What is your role as a clinic manager with respect to the CHW team?

V0.95; 12 September 2016

Probes:
Where team leader nurse is based in clinic
How much time do you expect the team leader to spend managing the CHW team?
Can you describe how she manages to balance her time between her clinic duties and the CHW team?
What happens when the team leader is not here?

Both where the team leader is based at the health post and the clinic
What engagement happens between you and CHW team leader? Between the CHW team and the clinic staff? What sort of things do they communicate about?

What have you been able to achieve with CHW?

Probes:
What are the successes the CHW team have been able to achieve?

What are the challenges?

Probes:
Have you ever had a complaint from the community about a CHW? What happened?

How do you engage with the community?

Probes:
What do the community think of the community health workers?
How does the community express their views?
Are there other organisations providing health care in the community and what do they do?
What is the role of the clinic committee? How effective is it?
Are training and tasks of the CHWs in line with local needs?

Ending the interview
Thank you. I think we've covered all of the questions that I wanted to ask you today. Do you have anything else you wanted to add?

Topics we would like to hear about from the interviewees:
recent health campaigns and role of community health workers;
access to supplies, transport, advice, equipment, space to meet and store;
referral management and feedback;
language barriers for community health workers; safety and security of CHW in the community;
previous training and experience of CHW;
other duties of nurse/manager;
incentives for the CHW;
complaints against a CHW and what happened.

Suggested generic prompts to use to encourage participants to continue:

To get going on a topic if they are unsure of where to start or what to say:
Start wherever you want
Start with what you think is most important
Why not start by telling me what happens in a day, starting at the beginning

To go into more depth or to encourage continuation of their account

Please tell me more about that
Please explain that for me
Why was that?
What did you feel about that?
What happened then?
Please can you tell me more about (person, role, place, organisation, activity etc.)

Annex B: Information Sheet for interviewing Nurse team leader of the CHW team

Formal Title: Implementing comprehensive, integrated, community-based health care for under-served, vulnerable communities in South Africa: A practical, evidence-informed model.

Hello, and thank you for your time today.

My name is ________. I work for the Centre for Health Policy at the University of the Witwatersrand. I am a researcher in the Batlhokomedi project, and I would like to interview you today as part of this project.

What is this research about?
We are conducting this research to understand how community health worker / WBOT programmes operate. We know that there are some innovative WBOT programmes in Sedibeng and we are keen to learn more about your programmes here. I believe you are the leader of a WBOT team. So we would like to hear about your experiences of leading a team of CHWs, both the successes and challenges.

The interview should take about 40 mins.

Voluntary Participation
It is up to you to decide whether you want participate in this study. If you agree to help with this research, and later change your mind, you are free to end the interview at any time, without any consequences.

Risks
There are no risks associated with talking to me today.

Confidentiality
No one other than my team members will be allowed to see the record of the interview discussion. We will not use your name in any reports of this work. We will use a code instead of your name e.g. A or B.

My notes and audio recordings will be kept on a secure university computer server. Participant codes will be kept in a locked filing cabinet under the care of the project site manager, and will be destroyed once our research is complete.

Approval for and benefits of this work
The study has been approved by the ethics committee of University of the Witwatersrand, and Gauteng Province. We hope the study will contribute new ideas and insights regarding the provision of community health worker services.

If you agree to be interviewed, we would also like your permission to record the interview as it really helps later in the office.

V0.95; 12 September 2016

What if I have any questions?
You are free to ask me any question about this research. If you have any further questions about the study, you are free to contact Julia De Kadt on Julia.DeKadt2@wits.ac.za, or 011-717-3434 or 074 336 9411.

If you are concerned about anything to do with the study in general, or wish to make a complaint, you can contact the chair of the ethics committee at the University of the Witwatersrand, who is Professor Peter Cleaton-Jones at telephone number: (011) 717-2301

Annex B Field version Nurse team leader of CHW interviews

Introduction

Thank you again for agreeing to be interviewed. Are you happy to start?

First, could you please tell me a bit about yourself?
Probes:
How many years have you worked as a nurse?
What is your professional title? (eg: Enrolled nurse, Professional nurse, other specialized nurse)
How many years have you worked at this facility?
How many years in the current position?
Were you born in this district?

Perhaps we could talk now about the Ward Based Outreach Team. Please tell me about how the Ward Based Outreach Team is run?
Probes:
How are community health workers recruited to the team?
Are there different types of CHW, with different training?
What resources are provided to CHW?
What happens in a typical day of the team?
How is the team's work for the day planned?
What happens on different days in the week?
How often do CHWs work after hours on weekends, and what do they do then?
How is work distributed within the team? Is there any division of labour? Do any CHWs have a leadership role within the team?
How does the data reporting system work?

- **What information do CHWs record while out in the community?**
- **What is done with this information? When?**
- **What forms are used?**
- **What is your role in the data reporting system? What exactly do you do?**

How does the referral system work?
Are there language barriers for WBOT?
Are there safety and security issues when working in the community? If so, what precautions do you take to ensure the safety of the CHW? What incidents are you aware of happening to the WBOT?
What incentives are there for the WBOT?

We would expect the following activities to be mentioned: advice giving to households; registration of households, case finding; referral management and feedback; recent health campaigns and role of community health workers.

What are your roles and responsibilities for WBOT?

Probes:

V0.95; 12 September 2016

What happens in a typical day for you?
What happens on different days in the week?
What happens when you are away?
What other responsibilities do you have at work, other than WBOT? Roughly, how much of your time do those activities take?
Please tell me about times when you have faced conflicting demands?
What engagement happens between you and manager and other staff at the clinic?
What engagement happens between the CHW team and the clinic staff?
What support do you receive for your work from colleagues ?

- **From District management**

What resources – financial, equipment, supplies, place to work, place to meet and store, transport, airtime

Do you receive any support from the community ?

What additional support would be helpful?

What dilemmas do you face in your day to day work?

What have you been able to achieve with WBOT?
Probes:
What are you proud of with respect to the achievements of WBOT?
What do you think are the key factors in achieving this?
What would enable you to achieve more?

What are the challenges?
Probes:
What challenges do you experience?
What do you do if one of the CHW is not doing their job very well?
Have there been any complaints against WBOT? What was it about? What happened?
Can you tell us anything about the strike earlier this year?

- **What happened?**
- **Have the issues that caused the strike been addressed?**

How do you engage with the community?
Probes:
What do the community think of the community health workers?
How does the community express their views?
Are training and services of CHWs well matched to local needs?
Can you tell me about the clinic committee? What sort of things do they engage with you about ?
Are there other organisations providing health care in the community? What do they do?
How do you work with these organisations?

Ending the interview
Thank you. I think we've covered all of the questions that I wanted to ask you today. Do you have anything else you wanted to add?

Suggested generic prompts to use to encourage participants to continue:
To get going on a topic if they are unsure of where to start or what to say:
Start wherever you want
Start with what you think is most important

V0.95; 12 September 2016

Why not start by telling me what happens in a day, starting at the beginning

To go into more depth or to encourage continuation of their account
Please tell me more about that
Please explain that for me
Why was that?
What did you feel about that?
What happened then?
Please can you tell me more about (person, role, place, organisation, activity etc.)

Annex C: Information Sheet for observing community health workers

Formal Title: Implementing comprehensive, integrated, community-based health care for under-served, vulnerable communities in South Africa: A practical, evidence-informed model.

Hello and thank you for your time today. My name is ________. I work for the Centre for Health Policy at the University of the Witwatersrand. I am a researcher in the Batlhokomedi project, and I would like to observe you today as part of this project.

What is this research about?
We are conducting this research to understand how community health worker / WBOT programmes operate. We know that there are some innovative WBOT programmes in Sedibeng and we are keen to learn more about your programmes here. I know you are a community health worker, so I would like to observe what you do as a community health worker. Without disrupting your work, I would also like to ask you about what you are doing while we are observing.

If you agree, while we are observing you and talking to you, we will take notes and then type these notes up.

<u>Voluntary Participation</u>
It is up to you to decide whether you want participate in this study. If you agree to help with this research, and later change your mind, you are free to withdraw at any time, without any consequences.

<u>Risks</u>
There are no risks associated with participating in the research.

<u>Confidentiality</u>
The information will be used for research purposes only. No one other than my team members will be allowed to see the record of the observation. We will not use your name in any reports of this work. We will use a code instead of your name e.g. A or B.

My notes will be kept on a secure university computer server, and participant codes will be kept in a locked filing cabinet under the care of the project site manager, and will be destroyed once our research is complete.

<u>Approval for and benefits of this work</u>

V0.95; 12 September 2016

The study has been approved by the ethics committee of University of the Witwatersrand, and Gauteng Province. The study will contribute new ideas and insights regarding the provision of community health worker services.

What if I have any questions?
You are free to ask me any question about this research. If you have any further questions about the study, you are free to contact Julia de Kadt on julia.dekadt2@wits.ac.za or 011 717 3434 or 074 336 9411.

If you are concerned about anything to do with the study in general, or wish to make a complaint, you can contact the chair of the ethics committee at the University of the Witwatersrand, who is Professor Peter Cleaton-Jones at telephone number: (011) 717-2301

Annex D: Information Sheet for observing nurse leader of community health worker team

Formal Title: Implementing comprehensive, integrated, community-based health care for under-served, vulnerable communities in South Africa: A practical, evidence-informed model.

Hello, and thank you for your time today. My name is ________. I work for the Centre for Health Policy at the University of the Witwatersrand. I am a researcher in the Batlhokomedi project, and I would like to observe you today as part of this project.

What is this research about?
We are conducting this research to understand how community health worker / WBOT programmes operate. We know that there are some innovative WBOT programmes in Sedibeng and we are keen to learn more about your programmes here. I believe you are the leader of a WBOT team. So we would like to observe what you do as a nurse, and what activities you do to support the CHWs. Without disrupting your work, we would also to ask you about what you are doing while we are observing. We do not wish to observe any consultations with patients in the clinic or health post, although we wish to observe home visits if you conduct any.

If you agree, while we are observing you and talking to you, we will take notes and then later type these notes up.

Voluntary Participation

Participation in this study is voluntary. If you agree to help with this research and later change your mind you are free to withdraw at any time, without any consequences.

Risks

There are no risks associated with participating in the research.

Confidentiality

V0.95; 12 September 2016

The information will be used for research purposes only. No one other than my team members will be allowed to see the record of the observation. We will not use your name in any reports of this work. We will use a code instead of your name e.g. A or B.

My notes will be kept on a secure university computer server. Participant codes will be kept in a locked filing cabinet under the care of the project site manager, and will be destroyed once our research is complete.

Approval for and benefits of this work

The study has been approved by the ethics committee of University of the Witwatersrand, and Gauteng Province. The study will contribute new ideas and insights regarding the provision of community health worker services.

What if I have any questions?

You are free to ask me any question about this research. If you have any further questions about the study, you are free to contact Julia de Kadt on julia.dekadt2@wits.ac.za or 011 717 3434 or 074 336 9411.

If you are concerned about anything to do with the study in general, or wish to make a complaint, you can contact the chair of the ethics committee at the University of the Witwatersrand, who is Professor Peter Cleaton-Jones at telephone number: (011) 717-2301

Annex E: Information sheet for group interview with community health workers

Formal Title: Implementing comprehensive, integrated, community-based health care for under-served, vulnerable communities in South Africa: A practical, evidence-informed model.

Hello, and thank you for your time today.

My name is ________. I work for the Centre for Health Policy at the University of the Witwatersrand. I am a researcher in the Batlhokomedi project, and I would like to talk to you today as part of this project.

What is this research about?

We are conducting this research to understand how community health worker / WBOT programmes operate. We know that there are some innovative WBOT programmes in Sedibeng and we are keen to learn more about your programmes here. I know you are community health workers, so we would like to hear about your experiences of being a CHW, both the successes and challenges.

The group interview should take about 1 hour.

Voluntary Participation

It is up to you to decide whether you want participate in this study. If you agree to help with this research, and later change your mind, you are free to leave the group interview at any time, without any consequences.

Risks

There are no risks associated with talking to me today.

Confidentiality

No one other than my team members will be allowed to see the record of the discussion. We will not use your name in any reports of this work. My notes and audio recordings will be kept on a secure university computer, and will be destroyed once our research is complete. We (the researchers) will keep the discussion confidential, and we would like to ask all the participants to do the same. However, as researchers we cannot guarantee this.

Approval for and benefits of this work

The study has been approved by the ethics committee of University of the Witwatersrand, and Gauteng Province. We hope the study will contribute new ideas on how to improve community health worker programmes.

If you agree to participate in the group interview, we would also like your permission to record the interview as it really helps later in the office.

<u>What if I have any questions?</u>
You are free to ask me any question about this research. If you have any further questions about the study, you are free to contact Julia De Kadt on Julia.DeKadt2@wits.ac.za, or 011-717-3434 or 074 336 9411.

If you are concerned about anything to do with the study in general, or wish to make a complaint, you can contact the chair of the ethics committee at the University of the Witwatersrand, who is Professor Peter Cleaton-Jones at telephone number: (011) 717-2301

Tool E: Group interview with Community Health Worker team

This will usually take place during the working day of the Community Health Worker's working day after they have had refreshments provided by the research team. The venue will usually be a quiet room in the clinic or health post. The supervising nurse will not be present.

The facilitator and the scribe conducting the group interview will introduce themselves. The facilitator will facilitate the discussion and the scriber will take field notes that will complement the audio-recording (e.g. behaviours, interruptions). The facilitator will encourage discussion between the Community Health Workers about what works well or not and why.

V0.95; 12 September 2016

At the start of the session ask the participants to complete the questionnaire

Check questionnaires are complete (facilitator and scribe to provide assistance where necessary). Check with group that they are ready and turn on the audio recorder announcing that you are doing this.

Introduction

Thank you again for agreeing to be interviewed. To start the session I am going to ask each of you in turn to tell me how you came to join the team?

Go around every participant in turn

Thank you. I am now going to ask you about your work with WBOT. Please all help to answer the questions.

I would like you to tell me about what you do day to day.

Please tell me about how a typical day at work starts, what do you do?
What happens during a typical day?
What happens at the end of the day?

Do you plan your work? How?

How do you plan your work or decide which households you'll visit on a particular day? (do you do this with your colleague(s) who pairs up with you?)
How do you keep track of the households where you need to follow up on a patient?

Do you do different tasks on different days of the week? What are these?

Can you please tell us a bit more about household registration?

When do you do household registration?

How often do you do household registration? How long does it take?

Is there any form you use when doing household registration?

What questions do you ask? What do you focus on?

Is there more than one kind of household registration?

What happens to these forms afterwards? Do you use them later on?

What happens to the information you collect during household registration? (probe, are there new patients identified and assigned after household registration? How are they assigned?)

Can you please tell us a bit more about home based care work?

Do you do any home based care? When does it happen?

How much of your time do you spend doing home based care work?

Please can you describe for us the data reporting system here?

What information do you record when you are out in the community?
What do you do with this information, and when?
How do you prepare your statistics?

What challenges do you experience with preparing your statistics?

Thank you. Now please tell me about your pack.

What is in it?
When do you use the contents? What for?
How are the consumables (name them e.g. forms, dressings) replenished?
How is the equipment (name it e.g. glucometer, bp machine,) maintained in working order?

What other support do you get to make your work easier?

Material resources, e.g. airtime, a meeting place, a place to store your files? Are these sufficient? If not, please explain?

Support from colleagues, your team leader, the clinic staff, from the community?

What do you do when you need help?

What type of situations do you find when you need help?
What help is available?
How do you access the help?
Is the help you receive sufficient? Please explain.

What happens when a team member is struggling or can't visit all of their households, for example because of sickness?

How does the team work when one member can't do their job?

What achievements in your work are you proud of?

What do you think has enabled you to achieve this?
What would enable you to achieve more?

What are the challenges that you face?

What dilemmas do you face in your day to day work?
Please tell me about times when you have faced conflicting demands?
Possible probes:
- Workload
- Completing paper work
- Preparing statistics
- Payment, Clocking system
- Transport
- Appreciation, recognition
- Conflict with community
- Conflict with OTL or facility/health post staff
- Working after hours & over weekend (types of tasks; how often, how long)

Job security

Do you feel that your job is secure? Have you encountered or heard problems about contract renewal with SmartPurse?

What do you feel about your prospects for career development and promotions?

Are you worried that you might face job loss or layoff?

If you were laid off, are you worried that you would have difficulty finding a suitable job?

How do you feel about SmartPurse?

Please could you tell us a bit about the strike?
What happened?
Have the issues been resolved?

How do you relate to the community?
What does the community think of you as a team?
How does the community share complaints, concerns or compliments about the WBOT team with you?
Are there other organisations providing health care in the community and what do they do?
How do you relate to them?

For CHWs who were previously working for an NGO:
Has your work changed since you moved from the NGO to the WBOT team? If so, how?
Which people in the community were you looking after when you worked for the NGO?

What kind of tasks did you do when you worked for the NGO?

Who was supervising you when you worked for the NGO?

What resources did you have when you worked for the NGO?

What challenges did you experience when you worked for the NGO?

How is your work different now that you work in the WBOT team?

<u>Ending the focus group</u>
Thank you. I think we've covered all of the questions that I wanted to ask you today. Do you have anything else you wanted to add?

<u>Topics we would like to hear about from the interviewees:</u>
guidelines, policies and protocols;
registration of households, case finding;
home visit records, numbers, planning;
recent health campaigns and role of Community Health Workers;
access to supplies, transport, advice, equipment, space to meet and store;
referral management and feedback;
language barriers;
safety and security of CHW in the community;
previous training and experience of CHW;
current/recent in role training
payment ;

supervision from team leader;
households refusing entry;
liaising with clinic staff;
communication with other organisations providing care in the community.

Suggested generic prompts to use to encourage participants to continue:

To get going on a topic if they are unsure of where to start or what to say:
Start wherever you want
Start with what you think is most important
Why not start by telling me what happens in a day, starting at the beginning

To go into more depth or to encourage continuation of their account
Please tell me more about that
Please explain that for me
Why was that?
What did you feel about that?
What happened then?
Please can you tell me more about (person, role, place, organisation, activity etc.)

CHW Focus Group Discussion Questionnaire **FGD ID: ________________________**

1	Date of interview		
2	Name of Health post / clinic		
3	Age (Years)		
4	Gender (please circle)	Male	Female
5	How many years have you worked as a CHW?		
6	How many years have you worked as a CHW in this area?		
7	How many years have you been a part of this team?		
8	How many households are you responsible for ?		

Travelling from home to the clinic or health post

9	How long does it take you to get from your house to the clinic or health post where you are based?	______ hours _______ minutes
10	How do you mainly travel from your home to the clinic or health	Walk<br>Taxi

	post where you are based? Please circle, and describe if other	Other: _________________
11	If you travel from your home to the households you visit by taxi, please provide the fare, in Rands.	

Travel from your home to the households you care for

12	If you travelled directly from your home to a household you care for, on average how long would this take you?	______ hours _______ minutes
13	If you needed to travel directly from your home to a household you car for, what mode of transport would you mainly use?	Walk<br>Taxi<br>Other: _________________
14	If you would travel from your home to a household you care for by taxi, please provide the fare, in Rands.	

Education, training and other employment

15	What is your highest level of education (please circle)	Some primary school<br>Completed primary school<br>Some secondary school<br>Completed secondary school (passed matric)<br>Some tertiary education	
16	If you have completed some tertiary education, please describe		
17	Please describe any CHW training you have done		
18	Do you have any other job apart from this one? (Please circle)	Yes	No
19	If you do have another job, please describe this		

V0.95; 12 September 2016

Annex G: Information Sheet for interviewing referred householder

Formal Title: Implementing comprehensive, integrated, community-based health care for under-served, vulnerable communities in South Africa: A practical, evidence-informed model.

Hello, and thank you for your time today.

My name is ________. I work for the Centre for Health Policy at the University of the Witwatersrand. I am a researcher in the Batlhokomedi project, and visited your household about a month ago with a community health worker. I am here today just to follow up with you after that visit.

What is this research about?
We are conducting this research to understand how community health worker / WBOT programmes operate. We are here to learn more about the community health workers in Sedibeng, by talking to people like yourself about what the CHWs do.

The interview should take about 40 mins.

Voluntary Participation

It is up to you to decide whether you want to participate in this study. If you agree to help with this research, and later change your mind, you are free to end the interview at any time, without any consequences.

Risks

There are no risks associated with talking to me today.

Confidentiality

No one other than my team members will be allowed to see the record of the interview. No contents of the interview will be shared with the clinic or health post, or any Community Health Workers, including the Community Health Worker who brought me here. We will not use your name in any reports of this work. We will use a code instead of your name e.g. A or B. My notes and audio recordings will be kept on a secure university computer server. Participant codes will be kept in a locked filing cabinet under the care of the project site manager, and will be destroyed once our research is complete.

Approval for and benefits of this work

V0.95; 12 September 2016

The study has been approved by the ethics committee of University of the Witwatersrand, and Gauteng Province. We hope the study will contribute new ideas and insights regarding the provision of community health worker services.

If you agree to be interviewed, we would also like your permission to record the interview as it really helps later in the office.

What if I have any questions?

You are free to ask me any question about this research. If you have any further questions about the study, you are free to contact Julia De Kadt on Julia.DeKadt2@wits.ac.za, or 011-717-3434 or 074 336 9411.

If you are concerned about anything to do with the study in general, or wish to make a complaint, you can contact the chair of the ethics committee at the University of the Witwatersrand, who is Professor Peter Cleaton-Jones at telephone number: (011) 717-2301

Tool G: Interviews with referred householder

Introduction

Thank you again for agreeing to be interviewed. Are you happy to start?

First, could you please tell me a little bit about yourself?
Probes:
Age
Gender
Language spoken
Number of people in household
Are you working?
If yes, what type of work do you do?
What other sources of income are there in the household (child support grants, pension, disability)?

Thank you. Now, let's talk a little bit about the Community Health Worker's visit. Please tell me about what happened when the Community Health Worker came to your house.
Probes:
What happened?
Can you describe how you felt about the visit that day?
Were any other household members around?
If so, what did other household members say or do?
What did the CHW suggest you do (in terms of your health/the issue you were experiencing)?
What did the CHW say she would do (in terms of your health/the issue you were experiencing)?

If the CHW suggested the patient should do something:
Were you able to do what the CHW suggested you do?
Probes:
What did you actually do?

Why?
What made it easy or difficult to do what the Community Health Worker suggested?
How helpful did you find the Community Health Worker's suggestion?

For householders who have not taken action based on the Community Health Worker recommendation:
Why didn't you do what the community health worker suggested?

If the CHW said she would do something:
Did the CHW do what she said she would do?
If the CHW didn't do what she said she would, do you know why? Do you think she will still do what she said?
Did the CHW do anything else?
Has the CHW made any other visits/follow-ups after the visit we've been discussing?
If so, when, and what did she do during those visits?

Could you please tell me about how you are doing now (with regards to your health/the issue you were experiencing)?
Probes (use only the appropriate ones):
What health care are you receiving now for your health?
Follow up visits, treatments, tests
What services are you receiving related to the issue you were experiencing?
Could we talk a bit about the CHW's service more generally?
Probes:
Is their service useful?
Can you give me any other examples of when they've assisted you?
Has it changed (over time)? How?
How could it be improved?
When did they start coming to see you?
How often do they usually come to see you?

Ending the interview
Thank you. I think we've covered all of the questions that I wanted to ask you today. Do you have anything else you wanted to add?

Suggested generic prompts to use to encourage participants to continue:

To get going on a topic if they are unsure of where to start or what to say:
Start wherever you want
Start with what you think is most important
Why not start by telling me what happens in a day, starting at the beginning

To go into more depth or to encourage continuation of their account
Please tell me more about that
Please explain that for me
Why was that?
What did you feel about that?
What happened then?
Please can you tell me more about (person, role, place, organisation, activity etc.)
