# Supplementary material for: Community health workers’ efforts to build health system trust in marginalised communities: a qualitative study from South Africa
Source: BMJ Open. 2021 May 19;11(5):e044065. doi: 10.1136/bmjopen-2020-044065 (PMC8137175; doi:10.1136/bmjopen-2020-044065)
Supplement: Author's manuscript [file bmjopen-2020-044065.draft_revisions.pdf]

BMJ Open is committed to open peer review. As part of this commitment we make the peer review history of every article we publish publicly available.

When an article is published we post the peer reviewers' comments and the authors' responses online. We also post the versions of the paper that were used during peer review. These are the versions that the peer review comments apply to.

The versions of the paper that follow are the versions that were submitted during the peer review process. They are not the versions of record or the final published versions. They should not be cited or distributed as the published version of this manuscript.

BMJ Open is an open access journal and the full, final, typeset and author-corrected version of record of the manuscript is available on our site with no access controls, subscription charges or pay-per-view fees (http://bmjopen.bmj.com).

If you have any questions on BMJ Open's open peer review process please email info.bmjopen@bmj.com

BMJ Open

# Community health workers' efforts to build health system trust in marginalised communities: A qualitative study from South Africa

| | |
|---|---|
| Journal: | *BMJ Open* |
| Manuscript ID | bmjopen-2020-044065 |
| Article Type: | Original research |
| Date Submitted by the Author: | 21-Aug-2020 |
| Complete List of Authors: | Anstey Watkins, Jocelyn; University of Warwick, Warwick Medical School<br>Griffiths, Frances; University of Warwick, Warwick Medical School; University of the Witwatersrand School of Public Health, Centre for Health Policy<br>Goudge, Jane; University of the Witwatersrand School of Public Health, Centre for Health Policy |
| Keywords: | QUALITATIVE RESEARCH, PUBLIC HEALTH, PRIMARY CARE |
| | |

SCHOLARONE™
Manuscripts

*I, the Submitting Author has the right to grant and does grant on behalf of all authors of the Work (as defined in the below author licence), an exclusive licence and/or a non-exclusive licence for contributions from authors who are: i) UK Crown employees; ii) where BMJ has agreed a CC-BY licence shall apply, and/or iii) in accordance with the terms applicable for US Federal Government officers or employees acting as part of their official duties; on a worldwide, perpetual, irrevocable, royalty-free basis to BMJ Publishing Group Ltd ("BMJ") its licensees and where the relevant Journal is co-owned by BMJ to the co-owners of the Journal, to publish the Work in this journal and any other BMJ products and to exploit all rights, as set out in our licence.*

*The Submitting Author accepts and understands that any supply made under these terms is made by BMJ to the Submitting Author unless you are acting as an employee on behalf of your employer or a postgraduate student of an affiliated institution which is paying any applicable article publishing charge ("APC") for Open Access articles. Where the Submitting Author wishes to make the Work available on an Open Access basis (and intends to pay the relevant APC), the terms of reuse of such Open Access shall be governed by a Creative Commons licence – details of these licences and which Creative Commons licence will apply to this Work are set out in our licence referred to above.*

*Other than as permitted in any relevant BMJ Author's Self Archiving Policies, I confirm this Work has not been accepted for publication elsewhere, is not being considered for publication elsewhere and does not duplicate material already published. I confirm all authors consent to publication of this Work and authorise the granting of this licence.*

For peer review only

# TITLE PAGE

**Title:** Community health workers' efforts to build health system trust in marginalised communities: A qualitative study from South Africa

**Authors:** Jocelyn Anstey Watkins[1], Frances Griffiths[1,2] and Jane Goudge[2]

## Affiliations
1. Warwick Medical School, University of Warwick, United Kingdom
2. Centre for Health Policy, School of Public Health, Faculty of Health Sciences, University of the Witwatersrand, Johannesburg, South Africa

**Corresponding author**
Prof Jane Goudge
Email: jane.goudge@gmail.com

**Word count** *(excluding title page, abstract, references, figures and tables/boxes)*: 5313

For peer review only

## ABSTRACT

**Introduction** Community health workers (CHWs) enable marginalised communities, often experiencing structural poverty, to access healthcare. Trust, important in all patient-provider relationships, is difficult to build in such communities, particularly when stigma associated with HIV/AIDS, TB, and now COVID-19, is widespread. CHWs, responsible for bringing people back into care, must repair trust. In South Africa, where a national CHW programme is being rolled out, marginalised communities have high levels of unemployment, domestic violence and injury.

**Objectives** In this complex social environment, we explored CHW workplace trust, interpersonal trust between the patient and CHW, and the institutional trust patients place in the health system.

**Design, Participants and Setting** Within the observation phase of a 3-year intervention study, we conducted interviews, focus groups and observations with patients, CHWs, their supervisors and, facility managers in Sedibeng.

**Results** CHWs had low levels of workplace trust. They had recently been on strike demanding better pay, employment conditions and, recognition of their work. They did not have equipment to perform work safely, and some colleagues did not trust, or value, their contribution. There was considerable interpersonal trust between CHWs and patients, however, CHWs' efforts were hampered by structural poverty, alcohol abuse, and no identification documents among long-term migrants. Those supervisors who understood the extent of the poverty supported CHW efforts to help the community. When patients had withdrawn from care, often due to nurses' insensitive behaviour, CHW's attempts to repair patient's institutional trust often failed due to the vulnerabilities of the community, and lack of support from the health system.

**Conclusion** Strategies are needed to build workplace trust with supportive supervision for CHWs and better working conditions, and to build interpersonal and institutional trust by ensuring sensitivity to social inequalities and the effects of structural poverty among healthcare providers. Societies need to care for everyone in them.


## ARTICLE SUMMARY

### Strengths and limitations

- The data was not collected to explicitly find out about trust. If it had been, participants may have expressed their views in greater detail.
- A strength of this research is the number of observational days that the fieldworkers spent with the community health workers and other staff to comprehend their daily work and community interactions.
- Our data illuminates the reality of the vulnerable communities lives in which CHWs work in - desperate poverty, alcoholism, and gender-based violence.
- The study was limited to a peri-urban South African setting, similar to other parts of South Africa and other LMIC contexts.

For peer review only

# MAIN TEXT

## INTRODUCTION

Community health workers (CHWs) serve a critical function in low- and middle-income countries (LMICs), providing frontline services to marginalised groups who face significant barriers to care[1-3]. Effective deployment of CHWs is crucial to moving towards the Sustainable Development Goals (SDG)[4,5]. In settings where HIV and tuberculosis (TB) are established epidemics, CHWs can assist people in adhering to treatment, important where drug resistance to antiretroviral treatment and TB are public health concerns[6,7].

In vulnerable communities, where people experience structural poverty[8], CHWs have to navigate complex health and social situations. Trust, important in all patient-provider-health system relationships[9], is more fragile in such communities[10]. Patients who have unstable lives often receive poorer care; in turn, poor quality care may cause patients to lose trust in their local facility, and become reluctant to seek care in the future[11]. CHWs, responsible for bringing people back into care, must repair that trust, a complex task when stigma associated with HIV/AIDS, TB and now COVID-19[12,13], is widespread.

In South Africa, a national CHW programme is being rolled out, particularly in marginalised communities with high levels of unemployment[14], domestic violence[15] and injury[16], an epidemic fuelled by high rates of alcohol abuse[17]. There is also a high prevalence of communicable and non-communicable conditions[18,19]. Patients struggle to stay in care due to poverty[20] and stigma,[21,22] and those who are migrants face inequity in the health system[23]. CHWs themselves are fighting for employment rights and recognition of their contribution, and so are challenging their relationship with the health system[24]. In this complex social environment, we explored CHW workplace trust, interpersonal trust between the patient and CHW, and the institutional trust patients place in the health system.

## BACKGROUND

### *CHW programme in South Africa*

In 2011, the South African Department of Health, initiated a national CHW programme (ward-based outreach teams; WBOTs) to improve access to services[25]. The intention is to provide health promotion, prevention, screening services and referral for a wide range of health and social needs[26]. The teams are composed of CHWs, supervised by one or two nurses, usually either a retired senior nurse (called a professional nurse) or junior nurse (called an enrolled nurse)[27]. Professional nurses (PNs) in South Africa can diagnose patients, prescribe treatment and dispense medication. PN supervisors are trained in primary healthcare and community nursing. Enrolled nurses (ENs) complete a two-year nursing course and are qualified to provide nursing care under supervision.

The standardised CHW training covers identification of the need for ante- and post-natal care, monitoring immunisation and adherence to chronic medication, screening for malnutrition and TB, substance abuse, and gender-based violence[28,29]. CHWs conduct household registrations to identify those in need, and trace patients who have withdrawn from care. During the COVID-19 pandemic, CHWs have been responsible for a mass community-based screening programme, asking people about symptoms and referring them to mobile testing units to quarantine suspected cases and provide appropriate care[30].

For peer review only

### *Trust*

Trust, a complex and multifaceted notion, "influences individuals' willingness to act on the basis of words, motives, intentions, actions and decisions of others under conditions of uncertainty, risk or vulnerability"[31]. The social relations of trust are accepted as a core contributor to health systems; a trust-based health system is grounded in cooperation, communication and empathy, enabling the successful functioning of the health service[32]. We have chosen to use the conceptual framework by Gilson et al.[33] which describes the interaction between workplace and patient-provider trust to frame our study (see Figure 1).

Workplace trust, defined as respectful and fair treatment in the workplace, is rooted in trust in the employing organisation, trust in your supervisor, and trust in your colleagues. Patient-provider trust is rooted firstly in interpersonal trust, in this case between a patient and CHW. Patient-provider trust is also rooted in institutional trust - the extent to which the CHW and patient can trust that the health system will support the CHWs to act in the best interests of the patient. Here the health system refers in an immediate sense to the CHW's supervisor, the facility manager, other staff at the facility, as well as the broader health system (access to equipment, medication, effectiveness of referrals to hospital, or social services). Both types of trust are influenced by the individual provider's characteristics, their personalities, past experiences, skills, knowledge, including, for example, sensitivity to patient concerns (e.g. stigma), ability to maintain confidentiality despite living in the same community, and to draw support from the health system.

## METHODS

### Study design

In the initial observation phase of a 3-year intervention study in Sedibeng District, Gauteng Province, we studied six CHW teams with different configurations of supervisors and locations. In analysing the observation data (reported elsewhere[34,35]), trust was a re-occurring theme. In this paper, we present the qualitative data from the four, non-intervention teams, to examine the role of trust in greater depth.

### Setting

In Sedibeng, at the time of the study, there were 39 CHW teams in 37 of the district's 72 wards (smallest geopolitical area). Sixteen of the teams were based at a health post and the remaining 23 were clinic-based. A health post consists of one or two temporary wooden structures (providing 3-6 rooms), without electricity and often with irregular water supply. It is managed by one or two PNs. In addition to the scope of practice described above, in Sedibeng the CHWs deliver chronic medication to elderly, or disabled, patients.

The CHWs were not formally employed by the government. They were an outsourced workforce and paid a minimal stipend for six-hours work per day. Two months before the start of our fieldwork, CHWs had been on strike over their conditions of employment. During the fieldwork, the CHWs were paid R2500 (ZAR), below the minimum wage (R3500 per month), however, by the end of 2018, it was increased to the minimum wage. All CHWs were from the local community.

The four teams served communities that differed by type of population and geography (See Box 1). Housing was either formal brick housing, or informal shacks often made from old corrugated iron, wood and plastic. Residents in the informal settlements are often internal migrants, from poor rural provinces, or long-term migrants, often second generation, originally from Lesotho without South African identification documentation[36], who cannot access government social grants or obtain employment.


For peer review only - http://bmjopen.bmj.com/site/about/guidelines.xhtml

**Box 1: Description of the teams and the communities**

**Team 1** – A clinic-based team supervised by a PN and two ENs; well-established, better-off community, with relatively low levels of unemployment. Many residents are elderly to whom the CHWs deliver medication.

**Team 2** – Team based at a health post and supervised by a PN and EN. Largely an informal settlement with very high levels of unemployment, with cases of extreme poverty and child malnutrition.

**Team 3** – Team based at health post supervised by a PN and two ENs; predominately formal brick housing, as well as an area with informal shacks.

**Team 4** – Health post team supervised by a PN and an EN; predominately government-built brick houses with back-room shacks, and a small informal settlement.

For peer review only

## Data collection

Interview and observation data were collected between September 2016 – February 2017 (Table 1). Fieldworkers (FW) were trained in research methods, ethics, and the study tools using extensive role-plays and observation practice. They were also given an orientation to community-based healthcare by an experienced nurse. Interview guides and observation templates were revised after piloting and feedback from the fieldworkers[35].

*Observations (obs) of CHWs* - CHWs were selected randomly by drawing names out of a hat, on the first morning of a four-day observation period. The fieldworkers observed 2-3 CHWs in the community, with or without a supervisor. The fieldworkers took detailed notes using a template.

*Focus group discussion* (FGD) topics included descriptions of the types of activities carried out by CHWs and their successes and challenges.

*Key informant interviews* were conducted with the facility manager (FM), and nurse supervisors (EN or PN) to discuss typical activities, resources, how the programme ran, and its successes and challenges.

*Patient interviews* - If during an observational visit a household member was given a referral by a CHW, the fieldworker asked their permission to conduct a follow-up interview one-month later to understand the patient's experience during any follow-up actions.

**Table 1: Qualitative data collected**

| Site | Team 1 | Team 2 | Team 3 | Team 4 |
|---|---|---|---|---|
| **Days of observations with CHWs (number of household visits)** | 24 (88) | 18 (43) | 26 (79) | 24 (65) |
| **Number of FGDs with CHWs (44 participants)** | 2 | 1 | 2 | 2 |
| **Number of key informant interviews (nurses and facility managers)** | 1 EN<br>1 PN | 1 EN<br>3 PNs<br>1 FM | 2 ENs<br>1 PN<br>1 FM | 1 EN<br>1 PN<br>1 FM |
| **Number of patient interviews** | 14 | 6 | 12 | 15 |



For peer review only - http://bmjopen.bmj.com/site/about/guidelines.xhtml

All participants were purposively sampled. All available CHWs participated in the FGDs and all nurse supervisors and facility managers were interviewed. None of the participants were known to the fieldworkers prior to the study. All data collection was conducted in person, audio-recorded and transcribed/translated verbatim by the fieldworkers who wrote reflective notes after each day. Patient interviews were summarised by the fieldworker. We deemed data saturation had been reached when each pair of CHWs had been observed for at least four-days.

### Data analysis

Raw data on trust was extracted from transcripts and notes into a data extraction table for each site by JAW. Data was placed chronologically so that the stories flowed from day-to-day and we could connect different participant's perspectives on the same issues. Familiarity with the extracted data enabled JAW, JG and FG to develop a common understanding about trust; based on this understanding, JAW identified a range of conceptual frameworks, and we chose Gilson et al.[33] as having the closest fit with the data.

We manually conducted a thematic analysis of the extracted data informed by the framework[33]. Emergent themes were identified and led us to adapt the framework. We compared data segments and quotes under each theme across the sites and between the participants in an iterative process of reflection.

Participants did not provide feedback on the findings, but one fieldworker was engaged to help check the interpretation of data. In the results we present this as a narrative, with anonymised participant quotations which are representative of each participant group and site.

### Ethics

All participants gave informed consent. The project received ethical approval from the University of the Witwatersrand Human Research Ethics Committee (Medical) (M160354), the Gauteng Provincial Health Research Committee, and from the University of Warwick Biomedical and Scientific Research Ethics Sub-Committee (REGO 2016-1825).

### Patient and Public Involvement

CHWs, patients and the public were not involved in the design, conduct, reporting or dissemination plans; however, facility, district and provincial managers were involved. We have conducted feedback sessions with CHW teams, facility, district and provincial managers.

## RESULTS

## Workplace Trust

### *Conditions of employment*

Many CHWs complained the stipend was not adequate to meet their basic needs and wanted fairer contracts with 21-days of annual leave, and employment after 50-years of age: "We feel that the Department of Health failed us. We were promised that we would be employed permanently, that our stipend would change to a salary." (CHW_FGD2-Team4) During the strike before fieldwork: "They started toyi-toying [protest dancing], singing the swearing songs at us [staff]. It was just havoc." (FM-Team2) At times, the strike was violent, corroding trust: "The CHWs attacked us and some of the staff were injured." (FM-Team2) Some CHWs continued a strategy of passive resistance: "They did not want to go to the community [to work]; some mentioned that they have nothing to eat at home… one said she will not come to work until she is paid." (FW_obs_CHW-Team2)



For peer review only - http://bmjopen.bmj.com/site/about/guidelines.xhtml

### *Working conditions*

The physical conditions at the health facilities were challenging. At the health post: "The patients wait outside and when it is raining, they get wet. In the consultation rooms, the nurses put buckets because the roof is leaking." (FW_obs_CHW-Team2) At the clinic, the supervisors complained: "We need space to work. It is frustrating because we work in the kitchen." (PN-Team1) The small spaces compromised infection control and made confidentiality difficult. Where there was no space inside, the CHW team met outside, limiting the possibility of discussing patients in confidence.

CHWs work in pairs for safety, even though the CHWs know their communities well: "Sometimes you go to the house with lots of males and we are scared that we might get raped. So, our lives are at risk." (CHW_FGD1-Team4) They were not given adequate personal protective equipment against TB. Other anxieties were not wearing gloves when caring for patients with bedsores, being pricked by a needle, or being harmed by dogs or people living with mental illness.

The CHWs were not supplied with a uniform; their supervisors suggested the CHWs wore black trousers and a white top to make them identifiable and look professional. The CHWs were given work bags containing equipment, however, glucose strips or batteries for the BP monitor were not routinely replaced: "It is so embarrassing when you are at the household, only to find out that the blood pressure machine is not working." (CHW_FGD1-Team3) The CHWs were told: "If they lose the equipment, they will have to replace it." (FW_obs_CHW-Team4) Their bags were routinely searched, and the content was ticked off an inventory by the clinic security guards: "as if they were thieves." (FW_obs _CHW-Team1) One PN threatened to stop their stipend if they did not return their bags each day, even though it was not within her power to do so.

### *Relationships with colleagues*

A hierarchy played out in the health facilities, with the CHWs on the bottom rung of the ladder: "Everyone tells us what to do. We have to do everything we are told to do without any question." (CHW_FGD-1-Team1) CHWs felt their work was not valued: "We have been working here for a long time and no one recognises us. We are just a group of fools." (CHW_FGD-1-Team2) One facility manager (FM) expressed this hierarchy: "The nurses are trained, they know exactly what they are supposed to do, unlike CHWs; they are just called from the streets." (FM-Team2) She justified her distrust: "When we send them out, there are those who will not be doing their work, they will go to their own places to do laundry and clean their houses." (FM-Team2) Her trust had been further eroded during the strike: "It is difficult for us to interact with them because they are very same people who had attacked us. We talk for the purposes of work, other than that, we are keeping them at arm's length." (FM-Team2) Another FM said that despite her grievances about them, "we learn to live with them and realise that these people are useful to us." (FM-Team4)

The CHW supervisors generally praised the CHWs. One EN talked of leading the CHWs, even while calling them children: "I know my children [CHWs]. It is important, you must be friendly and polite. Don't be a boss, be a leader. I talk to the CHWs. I tell them 'Be friendly… as I am'." (EN1-Team1) This attitude created a two-way relationship: "The CHWs are comfortable to raise issues with her. If they answer her, 'No sister [nurse], this should be like this and that', she listens to them, and allows them to take initiative and solve the problems." (FW_obs_PN- 1-Team4) Some of the ENs and PNs worked hard to reduce social hierarchy at the clinic, and ensure the CHW abilities were used constructively: "I feel that CHWs are not appreciated. I took my time and studied each CHW. Now I know how to handle each of them. If a CHW is rude, I bring her close to me and tell her that you will be doing statistics with me [collating activity data]." (PN1-Team1) This PN created leadership opportunities: "I said to her [CHW] you are their leader, if they have a problem, they must address it with you [CHW] and you will tell me [PN]." (PN1-Team1) Another PN tried to help the CHWs understand the importance of confidentiality and trusting relationships with patients: "Sometimes, they gossip


For peer review only - http://bmjopen.bmj.com/site/about/guidelines.xhtml

about their patients and that condemns the family. If they identify a TB patient and I would ask them to adopt that family." This was not always successful "[however] when I ask if they have delivered the treatment to the patient, [the CHW responds] 'why doesn't the patient come and fetch it?'." (PN2-Team2)

Some PNs viewed the CHWs as "unreliable. They just work for the sake of money… they aren't doing it wholeheartedly." (PN1-Team2) This PN did not trust they recorded the patient's blood pressure results accurately saying: "[CHWs] are not so honest." (PN1-Team2) One PN would belittle the CHWs: "She [PN] even shouts at us in front of the patients. We are adults, mothers and we have our own houses… she does not respect us." (CHW_FGD-1-Team3)

The less experienced ENs struggled to gain the respect of the CHWs: "I think it is because of my age maybe. They see me as young even though I am a nurse." (EN1-Team2) The CHWs relied on the PN, dismissing the EN's help: "The PN is much better, she was able to sit down with us and show us where we went wrong. The EN does not even sit with us. Last time there was a fight here they told her that, 'you are distant from us'." (CHW_FGD-1-Team2) The PN was aware this was a problem: "She [EN] has no comradeship with them. I can see that there are some [CHWs] who want to disrespect her because she is still very young." (PN1-Team2). One EN had little experience, and the CHW had to teach her: "The ENs know nothing about WBOT. We are the ones who had to teach them but they are getting more salary than us. That is not fair; that is why we get angry and strike." (CHW_FGD-Team4)

However, there was evidence of teamwork between the EN and CHWs: "We have two ENs who are able to walk to the field with us. They are helpful in intervening in our cases." (CHW_FGD-1-Team1) Another EN was happy to follow the CHWs' routine, helping when needed: "I don't change whatever they're doing that day, because they know their patients. I go and supervise wherever they are going. If they are having any problems, they will take me to those places so that I can help." (EN1-Team3) The supervisors were key to enlisting the support of other social services, even if their help was limited (Vignette 1).

**Vignette 1**

"I told the police there was a house written, 'no entry'. The police came and I got into the van and went with them. We found an old lady. There was a small bundle; she was sleeping on some blankets. It was winter. There bread, a towel, and porridge with nothing else. We bathed her and put on body lotion. After she bathed, she sat in the sun… she said, 'you remind of the days I used to bathe like this'. I gave her a blanket, towel, underwear, and nappies. I sat down and phoned SASSA [The South African Social Security Agency], and Home Affairs. When they checked her records, she was deported in 1975 back to Lesotho but she found a way to return to South Africa. Home Affairs said there is no way this woman can get an ID book. I am telling you about the problems the WBOT are experiencing." (PN2-Team2)

### *The community*

The CHWs are confronted with many complex, and at times, tragic situations: "The child's clinic card was lost. The shack caught fire and the family lost everything." (FW_obs_CHW-Team4) Community members were often intoxicated: "The CHWs find people sitting and drinking a Black Label [beer]. How do you talk to someone who is drinking liquor and talking nonsense?" (PN1-Team2) This made it difficult for the CHWs to do their jobs: "A patient said to us 'I am HIV positive but I don't take antiretrovirals. All I need is men and I survive'. The lady looked drunk. The CHW asked if she drinks alcohol and she said yes, she is trying to reduce stress." (FW_obs_CHW-Team3) The use of drugs was also apparent: "Some households, you find an old woman crying about her misbehaving grandson using nyaope [drugs]. You have to listen and give some counselling and leave when she is feeling



For peer review only - http://bmjopen.bmj.com/site/about/guidelines.xhtml

better." (FW_obs_CHW-Team1) Descriptions of physical abuse were not uncommon: "The granny started crying. The CHW gave her a tissue to wipe the tears. The granny said the man we passed outside is her husband, who abused her and made her leave home." (FW_obs_CHW-Team1) In another observed visit, when asked how she is doing, a patient replied saying: "she is stressed. She showed the CHW her bruises on her arms and said her husband is beating her." (FW_obs_CHW-Team4).

Many community members did not have legal documentation. With no South African identification, children's births are not registered nor are they eligible for government social grants: "The patient does not receive the pension grant because she is from Lesotho. Her son is also unemployed. The CHWs have tried to involve social workers and the police but there is no solution because of the documents." (FW_obs_CHW-Team2) This was a common occurrence, and CHWs are often not able to assist: "Our intervention is not enough because people are not working, those from Lesotho don't have identity documents. You are breaking their hearts because you can't give them anything, besides filling in the registration form and asking: 'Is there someone with TB? Is there someone working?,' when there is no food. You can't even say I will get food parcels, there is nothing." (CHW_FGD-1-Team2) Witnessing such poverty affected the CHWs: "We carry these stories because we are also human. I wish we could have one whole day just to talk about what we have seen and observed." (CHW_FGD-1-Team1)

## Interpersonal Trust (Patient - CHW)

There was a huge appreciation and respect for the work of the CHWs. "These people found me dying… I was not drinking water, not eating. The CHWs came every morning; they are ever-caring." (FW_obs_PN-Team4) Relying on the CHWs for emotional support was common: "I have found people that I can pour out my problem to… I feel very good after talking to them." (Patient-Team3) One man noticed that: "…we [CHWs] sit outside in the sun and he tried to erect a small shack so that we can sit there." (CHW_FGD-1-Team2) When the CHWs were on strike: "The community elders were even saying: 'if we were able to walk, we were going to join you [on the strike]'." (CHW_FGD-1-Team1) Gaining respect, and being acknowledged as a nurse, was hugely motivating: "I feel tall especially when they call me at the [shopping] mall saying 'Sister!'. Wow, I feel good'." (CHW_FGD-1-Team1)

Generally, the CHWs were sensitive to people's personal matters: "We do not tell the mother that her daughter did the HIV test. We can only be free to talk about it if the mother initiates the topic." (CHW_FGD-2-Team1) Home visits provided the opportunity to build supportive relationships: "I started working with the patient during her pregnancy after she was diagnosed with HIV/AIDS. She was devasted and unfortunately miscarried because of stress and depression. I continued to visit, and we developed a good relationship. The patient is taking treatment very well and her CD4 counts have improved." (FW_obs_CHW-Team4) On occasions, CHWs and nurses were not sensitive enough. "The EN asked the teenager if she is sexually active. The girl found the question very difficult to answer in front of her grandmother. One of the CHW's advised the EN to speak with the teenager in a private space. The EN took the advice and used the nearby kitchen to talk with the teenager." (FW_obs_CHW-Team1) The teenager was invited to the health post where she received sex education and contraceptives.

Some community members did not trust the CHWs. The PN supervisor had to reassure a patient that an HIV test would not be done by a CHW. Sometimes people do not answer their doors: "… because they fear that people would think they are HIV positive, but we are visiting everyone." (PN1-Team4) They were blamed for not delivering medications on time: "We go to their houses to deliver medications and they are not there. Then the patient will come to the clinic to complain. So, it



For peer review only - http://bmjopen.bmj.com/site/about/guidelines.xhtml

seems as if we don't do our work." (CHW_FGD-1-Team1) Occasionally, when the clinic does not have any medication, the CHWs were blamed for selling the medication.

## Institutional Trust (Patient – CHW – Nurse – Health system)

CHWs can make a formal referral to the health facility. In two sites, referred patients did not have to queue at the clinic, but could go straight to the CHW supervisor. The teenager's referral (above) enabled a streamlined visit for her first contraception visit: "The referral helped me because it was the first time I visited the clinic. It made it easy as I knew exactly who I was looking for." (PatientTeam1) However, without adequate support from the clinic, CHWs often struggled to support patients: "I had an incident where a patient who tested negative [HIV] throughout her pregnancy but on delivery tested positive. The clinic gave her antiretrovirals but she left them at the clinic. I took them to the woman and she told me, she won't take those them because she is not HIV positive. I had to do counselling which I am not qualified to perform." (CHW_FGD-Team4) The CHW efforts are sometimes frustrated by the clinic 'rules': "Some households do not have food to eat and the patient is on treatment. I contribute something [from my own pocket] so that the patient can eat, but when I ask for porridge from the clinic, the nurse tells me that I have to bring the patient [to the clinic]. That is a big problem because the patient is sick and has no transport money. So, yah it hurts." (CHW_FGD-1-Team3) The nurse was not willing to accept the CHW's word that the person cannot come, and failure to support a patient reduces the CHWs credibility in the community.

In other instances, there was a breakdown in communication with other parts of the system: "She used to go and collect her [government] grant by herself but for the previous two-months she has been bedridden. We promised her a wheelchair. We phoned the hospital, but it was not possible for us to take the old lady to the hospital because of the distance. I don't know what happened [after that]." (FW_obs_CHW-Team2) At times, the complex transitory nature of people's lives made helping them difficult: "A woman was breastfeeding and defaulted on her antiretrovirals, and was refusing to come to the clinic, so they were concerned about the safety of the baby. The CHW referred the patient to the social worker, but she does not think the social worker managed to find the patient because she disappeared." (PN3-Team2)

Some nurses were sensitive to the challenges that the communities faced. "When you sit down and talk with them you would find that they have buckets of stressors. I don't have tablets for stress. I have to try and talk to the person." (PN1-Team2) The same nurse was also aware that her privileged position made her insensitive to patients' challenges: "I asked this woman whether she had bathed or not, because the child was dirty and the woman was also untidy, and she said no. I really got embarrassed because she told me that there was no body soap because there was 'no one working at home, we are left with our grandmother, our mother is dead, and we are from Lesotho'." (PN1-Team2)

However, negative attitudes of nurses often affected the patient's willingness to attend the health facility: "The CHWs asked an old lady why she is not taking her treatment. The lady said the sister [nurse] at the clinic doesn't talk to her well, so it is better for her to stop going to the clinic." (FW_obs_CHW-Team3) Some patients confided in the CHWs about the nurses' behaviour: "[the patient] said if the nurse comes to their houses and speaks the way she speaks when she is at the clinic, they are going to hit her." (CHW_FGD-2-Team3) Vignette 2, describes a woman who was no longer taking her antiretroviral treatment due to a disagreement with the health post staff. In vignette 3, a nurse is rude to a vulnerable pregnant woman who withdraws from care; the CHW enlists the PN's support to get the nurse to apologise but fails.

| **Vignette 2** |
|---|

For peer review only

The CHW asked the woman why she is breastfeeding whilst she is HIV positive and not taking treatment: "She said she and her child are both fine, the child has never been sick, and she is picking up weight: 'I will see about that thing when I am sick'." (FW_obs_CHW-Team4) The nurse had taken bloods from her baby, but the results were lost. Before retesting the child, the woman was insisting: "The clinic must first tell me what happened to the blood that was drawn." (FW_obs_CHW-Team4)

**Vignette 3**

A patient was raped as a teenager and contracted HIV. Her first child passed away and her second child was also HIV positive. The patient usually takes antiretrovirals. She is pregnant. When visiting the clinic, a nurse refused to check the unborn baby because the mother did not have a transfer letter from her previous clinic in the Eastern Cape. The consultation ended with the nurse saying: "I don't care if you give birth in the toilet or in the street. It is not my problem." (FW_obs_CHW-Team3) Feeling angry and upset, the pregnant woman refused to go back to the clinic. With tears flowing down her face the woman said: "I know the rules of the clinic for a pregnant mother who is HIV positive, and I follow them always. I did not choose to be HIV positive." (Patient-Team3) The CHW promised to collect her medication on her behalf, and also she would report the case to the supervisor, saying: "she [patient] must not worry as she is not the first one she has treated badly and this time she [CHW] is going to report her." (FW_obs_CHW-Team3) However after the patient and nurse communicated, the patient reported: "the sister [nurse] denied all the things she said to me, they wrote on my clinic card that I will never come back to this clinic, and the clinic will not be accountable should anything happen to me." (Patient-Team3)

Responsible for finding defaulting patients, the CHWs have to find ways to repair the patient's trust in the health system: "We talk to the patients, encourage them to go for the sake of their health and ignore the nurses' attitude." (CHW_FGD-2-Team3) However, the CHWs could not guarantee patients will be treated better next time: "I [CHW] told the patient that when you [don't go to] the clinic, you are killing yourself as you are the one who is taking treatment, not the sister [nurse] and the life that you are living is yours but not the sister's." (CHW_FGD-1-Team3) A few people said they would only return to seek care if the CHW accompanied them: "Okay, I will come only if you [CHW] will be there too." (FW_obs_CHW-Team4)

## DISCUSSION

In this paper, we have explored both workplace trust, patient-CHW interpersonal trust and patient-health system (institutional) trust, amongst CHW teams in South Africa. The CHWs are the lowest cadre in the health system; their conditions of employment, their working environment, the lack of necessary equipment to perform their work safely, and the treatment by some colleagues indicated to the CHWs that their work was unrecognised, their contribution untrusted. The low levels of monetary incentives and poor working conditions of CHWs have been reported elsewhere in South Africa[37], and other resourced constrained settings[38]. By striking, the CHWs in our study were demanding better pay and employment conditions, as well as recognition of the importance of their work.

The CHWs are working in communities mired in complex social problems the result of long-term structural poverty. Dysfunctional family relationships impact on patients' ability to look after themselves and take their treatment. Alcohol abuse blights the lives of community members, making the work of the CHWs harder. The lack of identification documents among long-term migrants leads to desperate poverty that makes accessing healthcare difficult, even with the CHWs' efforts. Some supervisors understood the extent of the poverty in the surrounding communities,

For peer review only

from which the CHWs themselves came. These supervisors worked hard to overcome the social hierarchy by building the CHW's skills, and supporting their efforts to help the community.

There was considerable evidence of interpersonal trust between patients and CHWs, with many people appreciative of their work. CHW's operate in a unique environment, where household visits enable strong relationships to be built, but living in the same community can test the CHWs' ability to maintain confidentiality[10]. CHWs have to make sensitive judgements about when and what to ask people in order to build trust, a difficult terrain to navigate, particularly because of the vulnerability of many of their patients.

The attitude of some of the facility-based nurses ("bad apples"[39]) led some patients to withdraw from care[40]. Insensitive to stigma and barriers to accessing care that the socio-economic conditions of people's lives create, nurses' behaviour offended patients. CHW's attempts to repair trust often failed due to the vulnerabilities of the community, and lack of support from the health system, underpinned by poor workplace trust including CHWs often fraught relationships with their colleagues. We have reported elsewhere that inadequate and unpredictable support from the clinic negatively affects the CHW's ability to provide care and in turn, their credibility in the community[34].

Migrants, often unable to seek care because of their poverty, are being denied a fundamental human right. Section 27 of the 1996 South African Constitution states that "everyone has the right to have access to health services". The National Health Insurance (NHI) Bill[41] states that illegal and undocumented migrants "will receive basic healthcare services" (emergency care and treatment for HIV, TB and malaria), but not general primary healthcare or sexual and reproductive services[42]. It is not possible to provide effective HIV treatment without related primary healthcare services[43], and this is at odds with the SDG of 'leaving no one behind'[44] and achieving universal health coverage. Societies need to care for everyone in them.

## Limitations and strengths

The data was not collected to explicitly find out about trust. If it had been, participants may have expressed their views in greater detail. Our data illuminates the reality of the communities lives in which CHWs work in - desperate poverty, alcoholism, and gender-based violence. The study was limited to a peri-urban South African setting, similar to other parts of South Africa and other LMIC contexts.

## Recommendations

Given the interconnected nature of workplace, interpersonal and institutional trust, our recommendations include:

1. CHWs and nurses should be provided with opportunities to develop a better understanding of, and empathy for, the community's health and social situation.
2. Facility managers and nurses need to work to overcome social hierarchy in the facility, so CHWs feel supported in their workplace and patients feel cared for.
3. Inexperienced ENs need to be mentored while they develop as CHW supervisors.
4. In communities with complex social problems, the CHWs and their supervisors need strong intersectoral collaborations with other services.
5. Migrants need to have the right, and means, to be able to access care.

## Conclusion

CHWs' role in enabling vulnerable communities to access care is underpinned by workplace, interpersonal trust and institutional trust. Without these different forms of trust, CHWs struggle to assist patients to stay in care; yet creating trust in marginalised communities struggling with


For peer review only - http://bmjopen.bmj.com/site/about/guidelines.xhtml

structural poverty is far from easy. Nurses and CHW supervisors need to be sensitive to the hierarchy created by social inequalities and the barriers that patients face in accessing care. They need to support the CHWs in helping patients overcome these barriers. The government's role in ensuring migrants' rights to accessing healthcare services is crucial in developing trust.

**Acknowledgements**

The authors would like to thank the Sedibeng community and primary healthcare staff for being involved in the study. Thank you, also to the dedicated team of fieldworkers working for the Bathlokomedi project.

**Contributionship**

JG and FG are the principal investigators and award holders on this grant, conceptualising the study and managing data collection. They contributed to the supervision, data analysis and interpretation and drafting this article. JAW extracted and analysed the data and wrote the article, with JG editing drafts. All authors reviewed the final draft of this article.

**Funding**

The work was supported by the UK Medical Research Council [grant number MR/N015908/1].

**Competing Interests**

The authors declare that they have no financial or personal relationships that may have inappropriately influenced them in writing this article.

**Patient Consent for publication**

Obtained

**Data sharing statement**

Data is available upon request from Prof Jane Goudge (jane.goudge@gmail.com).

For peer review only

## REFERENCES

1. Kok MC, Ormel H, Broerse JE, et al. Optimising the benefits of community health workers' unique position between communities and the health sector: a comparative analysis of factors shaping relationships in four countries. *Global Public Health* 2017;12(11):1404-32. doi: 10.1080/17441692.2016.1174722
2. Daniels K, Odendaal WA, Nkonki L, et al. Incentives for lay health workers to improve recruitment, retention in service and performance. *The Cochrane Database of Systematic Reviews* 2019;2019(12) doi: 10.1002/14651858.CD011201
3. Ludwick T, Morgan A, Kane S, et al. The distinctive roles of urban community health workers in low-and middle-income countries: a scoping review of the literature. *Health Policy and Planning* 2020;czaa049 doi: doi.org/10.1093/heapol/czaa049
4. Wahl B, Lehtimaki S, Germann S, et al. Expanding the use of community health workers in urban settings: a potential strategy for progress towards universal health coverage. *Health Policy and Planning* 2020;35(1):91-101. doi: 10.1093/heapol/czz133



For peer review only - http://bmjopen.bmj.com/site/about/guidelines.xhtml

5. Maher D, Cometto G. Research on community-based health workers is needed to achieve the sustainable development goals. World Health Organisation. Geneva. 2016 [Available from: https://www.who.int/bulletin/volumes/94/11/16-185918/en/ accessed 29th July 2020.
6. Chimukangara B, Lessells RJ, Rhee S-Y, et al. Trends in pretreatment HIV-1 drug resistance in antiretroviral therapy-naive adults in South Africa, 2000–2016: a pooled sequence analysis. *EClinicalMedicine* 2019;9:26-34. doi: 10.1016/j.eclinm.2019.03.006
7. Streicher EM, Müller B, Chihota V, et al. Emergence and treatment of multidrug resistant (MDR) and extensively drug-resistant (XDR) tuberculosis in South Africa. *Infection, Genetics and Evolution* 2012;12(4):686-94. doi: 10.1016/j.meegid.2011.07.019
8. Rylko-Bauer B, Farmer P. Structural violence, poverty, and social suffering. *The Oxford Handbook of the Social Science of Poverty* 2016:47-74.
9. Brennan N, Barnes R, Calnan M, et al. Trust in the health-care provider–patient relationship: a systematic mapping review of the evidence base. *International Journal for Quality in Health Care* 2013;25(6):682-88. doi: 10.1093/intqhc/mzt063
10. Grant M, Wilford A, Haskins L, et al. Trust of community health workers influences the acceptance of community-based maternal and child health services. *African Journal of Primary Health Care & Family Medicine* 2017;9(1):1-8. doi: 10.4102/phcfm.v9i1.1281
11. Eyles J, Harris B, Fried J, et al. Suspicious minds: Apportioning and avoiding blame for distrustful relationships and deferring medical treatment in South Africa. *Sociology Mind* 2015;5(03):188. doi: 10.4236/sm.2015.53017
12. Besada D, Daviaud E. If we invested in this today, South Africa could save billions — and fight COVID-19. Bhekisisa Centre for Health Journalism. South Africa. 2020 [1st July 2020]. Available from: https://bhekisisa.org/opinion/2020-05-20-community-health-care-workers-in-south-africa-investment-case-covid19-coronavirus-tracing-programme/ accessed 1st July 2020.
13. Xolo N. Concerns mount over Covid-19 stigma in KZN. Maverick Citizen South Africa, 2020 [Available from: https://www.dailymaverick.co.za/article/2020-05-25-concerns-mount-over-covid-19-stigma-in-kzn/#gsc.tab=0 accessed 22nd July 2020.
14. Fransman T, Yu D. Multidimensional poverty in South Africa in 2001–16. *Development Southern Africa* 2019;36(1):50-79. doi: 10.1080/0376835X.2018.1469971
15. Meyiwa T, Williamson C, Maseti T, et al. A twenty-year review of policy landscape for gender-based violence in South Africa. *Gender and Behaviour* 2017;15(2):8607-17.
16. Seedat M, Van Niekerk A, Jewkes R, et al. Violence and injuries in South Africa: prioritising an agenda for prevention. *The Lancet* 2009;374(9694):1011-22. doi: 10.1016/S0140-6736(09)60948-X
17. Herrick C, Parnell S. Alcohol, poverty and the South African city. *South African Geographical Journal* 2014;96(1):1-14. doi: 10.1080/03736245.2014.896277
18. Oni T, Youngblood E, Boulle A, et al. Patterns of HIV, TB, and non-communicable disease multi-morbidity in peri-urban South Africa-a cross sectional study. *BMC Infectious Diseases* 2015;15(1):1-8. doi: 10.1186/s12879-015-0750-1
19. Mayosi BM, Flisher AJ, Lalloo UG, et al. The burden of non-communicable diseases in South Africa. *The Lancet* 2009;374(9693):934-47. doi: 10.1016/S0140-6736(09)61087-4
20. Goudge J, Gilson L, Russell S, et al. Affordability, availability and acceptability barriers to health care for the chronically ill: longitudinal case studies from South Africa. *BMC Health Serv Res* 2009;9:75. doi: 10.1186/1472-6963-9-75
21. Kalichman SC, Mathews C, Banas E, et al. Treatment adherence in HIV stigmatized environments in South Africa: stigma avoidance and medication management. *International Journal of STD & AIDS* 2019;30(4):362-70. doi: 10.1177/0956462418813047
22. Mabunda K, Ngamasana EL, Babalola JO, et al. Determinants of poor adherence to antiretroviral treatment using a combined effect of age and education among human immunodeficiency virus infected young adults attending care at Letaba Hospital HIV Clinic, Limpopo Province,


For peer review only - http://bmjopen.bmj.com/site/about/guidelines.xhtml

South Africa. *The Pan African Medical Journal* 2019;32 doi: 10.11604/pamj.2019.32.37.17722

23. Vearey J, Modisenyane M, Hunter-Adams J. Towards a migration-aware health system in South Africa: A strategic opportunity to address health inequity. *South African Health Review* 2017;2017(1):89-98.
24. Trafford Z, Swartz A, Colvin CJ. "Contract to Volunteer": South African community health worker mobilization for better labor protection. *New Solutions: A Journal of Environmental and Occupational Health Policy* 2018;27(4):648-66. doi: 10.1177/1048291117739529
25. SADoH. Provincial guidelines for the implementation of the three streams of PHC Re-engineering: South African Department of Health; 2011 [Available from: http://www.jphcf.co.za/wp-content/uploads/2014/06/GUIDELINES-FOR-THE-IMPLEMENTATION-OF-THE-THREE-STREAMS-OF-PHC-4-Sept-2.pdf accessed 19th August 2020.
26. Schneider H, Schaay N, Dudley L, et al. The challenges of reshaping disease specific and care oriented community based services towards comprehensive goals: a situation appraisal in the Western Cape Province, South Africa. *BMC Health Services Research* 2015;15(1):436. doi: 10.1186/s12913-015-1109-4
27. Mhlongo EM, Lutge E. The roles, responsibilities and perceptions of community health workers and ward-based primary health care outreach teams (WBPHCOTs) in South Africa: a scoping review protocol. *Systematic Reviews* 2019;8(1):1-7. doi: 10.1186/s13643-019-1114-5
28. Griffiths F, Babalola O, Brown C, et al. Development of a tool for assessing quality of comprehensive care provided by community health workers in a community-based care programme in South Africa. *BMJ Open* 2019;9(9):e030677. doi: 10.1136/bmjopen-2019-030677
29. SAQA. National Certificate: Community Health Work Phase 1 and 2. South African Qualifications Authority 2018 [Available from: https://qspe.saqa.org.za/showQualification.php?id=64749 accessed 10th August 2020.
30. Daniel L. Coronavirus: what is South Africa COVID home-visits-program South Africa, 2020 [Available from: https://www.thesouthafrican.com/news/coronavirus-what-is-south-africa-covid-home-visits-program/ accessed 22nd July 2020.
31. Okello DR, Gilson L. Exploring the influence of trust relationships on motivation in the health sector: a systematic review. *Human Resources for Health* 2015;13(1):16. doi: 10.1186/s12960-015-0007-5
32. Gilson L. Trust and the development of health care as a social institution. *Social Science & Medicine* 2003;56(7):1453-68. doi: 10.1016/S0277-9536(02)00142-9
33. Gilson L, Palmer N, Schneider H. Trust and health worker performance: exploring a conceptual framework using South African evidence. *Social Science & Medicine* 2005;61(7):1418-29. doi: 10.1016/j.socscimed.2004.11.062
34. Tseng Y-h, Griffiths F, de Kadt J, et al. Integrating community health workers into the formal health system to improve performance: a qualitative study on the role of on-site supervision in the South African programme. *BMJ Open* 2019;9(2):e022186. doi: 10.1136/bmjopen-2018-022186
35. Goudge J, Kadt Jd, Babalola O, et al. Household coverage, quality and costs of care provided by community health worker teams and the determining factors: Findings from a mixed methods study in South Africa. *BMJ Open* 2020;10:e035578. doi: 10.1136/bmjopen-2019-035578
36. Segatti A, Landau L. Contemporary migration to South Africa: a regional development issue: The World Bank 2011.
37. Maboko S, Hlongwana K, Mashamba-Thompson TP. Factors influencing the motivation of community health workers in Vhembe district, Limpopo: case study. *South African Health Review* 2018;2018(1):67-68.

For peer review only

38. Singh D, Negin J, Otim M, et al. The effect of payment and incentives on motivation and focus of community health workers: five case studies from low-and middle-income countries. *Human Resources for Health* 2015;13(1):1-12. doi: 10.1186/s12960-015-0051-1
39. Kane S, Calnan M, Radkar A. Trust and trust relations from the providers' perspective: the case of the healthcare system in India. *Indian Journal of Medical Ethics* 2015;12(3):157-68. doi: 10.20529/IJME.2015.045
40. Nkosi B, Seeley J, Ngwenya N, et al. Exploring adolescents and young people's candidacy for utilising health services in a rural district, South Africa. *BMC Health Services Research* 2019;19(1):195. doi: 10.1186/s12913-019-3960-1
41. NDOH. South African National Health Insurance White Policy Paper. *The National Department of Health, South Africa* 2017
42. Section27. Spotlight on NHI: NHI Bill will divert SA from Universal Health Coverage if some groups are excluded. South Africa, 2019 [Available from: http://section27.org.za/2019/11/spotlight-on-nhi-nhi-bill-will-divert-sa-from-universal-health-coverage-if-some-groups-are-excluded/ accessed 6th August 2020.
43. Faturiyele I, Karletsos D, Ntene-Sealiete K, et al. Access to HIV care and treatment for migrants between Lesotho and South Africa: a mixed methods study. *BMC Public Health* 2018;18(1):668. doi: 10.1186/s12889-018-5594-3
44. Stuart E, Woodroffe J. Leaving no-one behind: can the Sustainable Development Goals succeed where the Millennium Development Goals lacked? *Gender & Development* 2016;24(1):69-81. doi: 10.1080/13552074.2016.1142206

For peer review only

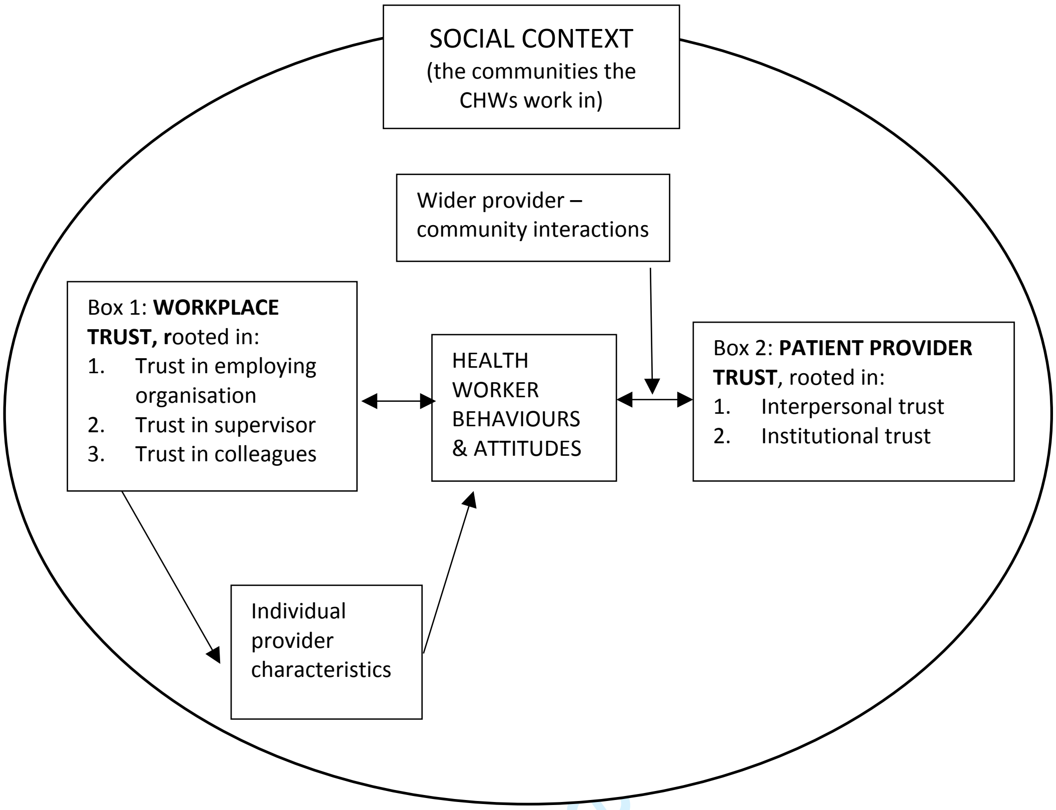


**Figure 1. Conceptual framework adapted from Gilson et al., 2005**

eview only

## COREQ (COnsolidated criteria for REporting Qualitative research) Checklist

A checklist of items that should be included in reports of qualitative research. You must report the page number in your manuscript where you consider each of the items listed in this checklist. If you have not included this information, either revise your manuscript accordingly before submitting or note N/A.

| Topic | Item No. | Guide Questions/Description | Reported on Page No. |
|---|---|---|---|
| **Domain 1: Research team and reflexivity** | | | |
| *Personal characteristics* | | | |
| Interviewer/facilitator | 1 | Which author/s conducted the interview or focus group? | 5 |
| Credentials | 2 | What were the researcher's credentials? E.g. PhD, MD | |
| Occupation | 3 | What was their occupation at the time of the study? | |
| Gender | 4 | Was the researcher male or female? | |
| Experience and training | 5 | What experience or training did the researcher have? | |
| *Relationship with participants* | | | |
| Relationship established | 6 | Was a relationship established prior to study commencement? | 6 |
| Participant knowledge of the interviewer | 7 | What did the participants know about the researcher? e.g. personal goals, reasons for doing the research | |
| Interviewer characteristics | 8 | What characteristics were reported about the inter viewer/facilitator? e.g. Bias, assumptions, reasons and interests in the research topic | |
| **Domain 2: Study design** | | | |
| *Theoretical framework* | | | |
| Methodological orientation and Theory | 9 | What methodological orientation was stated to underpin the study? e.g. grounded theory, discourse analysis, ethnography, phenomenology, content analysis | 6 |
| *Participant selection* | | | |
| Sampling | 10 | How were participants selected? e.g. purposive, convenience, consecutive, snowball | 6 |
| Method of approach | 11 | How were participants approached? e.g. face-to-face, telephone, mail, email | 6 |
| Sample size | 12 | How many participants were in the study? | 6 |
| Non-participation | 13 | How many people refused to participate or dropped out? Reasons? | 6 |
| *Setting* | | | |
| Setting of data collection | 14 | Where was the data collected? e.g. home, clinic, workplace | 5 |
| Presence of non-participants | 15 | Was anyone else present besides the participants and researchers? | 6 |
| Description of sample | 16 | What are the important characteristics of the sample? e.g. demographic data, date | 5 |
| *Data collection* | | | |
| Interview guide | 17 | Were questions, prompts, guides provided by the authors? Was it pilot tested? | 6 |
| Repeat interviews | 18 | Were repeat inter views carried out? If yes, how many? | |
| Audio/visual recording | 19 | Did the research use audio or visual recording to collect the data? | 6 |
| Field notes | 20 | Were field notes made during and/or after the inter view or focus group? | 6 |
| Duration | 21 | What was the duration of the inter views or focus group? | |
| Data saturation | 22 | Was data saturation discussed? | 6 |
| Transcripts returned | 23 | Were transcripts returned to participants for comment and/or | 7 |

For peer review only

| Topic | Item No. | Guide Questions/Description | Reported on Page No. |
|---|---|---|---|
| | | correction? | |
| **Domain 3: analysis and findings** | | | |
| *Data analysis* | | | |
| Number of data coders | 24 | How many data coders coded the data? | 6 |
| Description of the coding tree | 25 | Did authors provide a description of the coding tree? | 6 |
| Derivation of themes | 26 | Were themes identified in advance or derived from the data? | 6 |
| Software | 27 | What software, if applicable, was used to manage the data? | 6 |
| Participant checking | 28 | Did participants provide feedback on the findings? | 7 |
| *Reporting* | | | |
| Quotations presented | 29 | Were participant quotations presented to illustrate the themes/findings? Was each quotation identified? e.g. participant number | 7-12 |
| Data and findings consistent | 30 | Was there consistency between the data presented and the findings? | 7-12 |
| Clarity of major themes | 31 | Were major themes clearly presented in the findings? | 7-12 |
| Clarity of minor themes | 32 | Is there a description of diverse cases or discussion of minor themes? | 12 |

Developed from: Tong A, Sainsbury P, Craig J. Consolidated criteria for reporting qualitative research (COREQ): a 32-item checklist for interviews and focus groups. *International Journal for Quality in Health Care*. 2007. Volume 19, Number 6: pp. 349 – 357

**Once you have completed this checklist, please save a copy and upload it as part of your submission. DO NOT include this checklist as part of the main manuscript document. It must be uploaded as a separate file.**

For peer review only

BMJ Open

# Community health workers' efforts to build health system trust in marginalised communities: A qualitative study from South Africa



| | |
|---|---|
| Journal: | *BMJ Open* |
| Manuscript ID | bmjopen-2020-044065.R1 |
| Article Type: | Original research |
| Date Submitted by the Author: | 20-Feb-2021 |
| Complete List of Authors: | Anstey Watkins, Jocelyn; University of Warwick, Warwick Medical School<br>Griffiths, Frances; University of Warwick, Warwick Medical School; University of the Witwatersrand School of Public Health, Centre for Health Policy<br>Goudge, Jane; University of the Witwatersrand School of Public Health, Centre for Health Policy |
| <b>Primary Subject Heading</b>: | Health services research |
| Secondary Subject Heading: | Global health |
| Keywords: | QUALITATIVE RESEARCH, PUBLIC HEALTH, PRIMARY CARE, International health services < HEALTH SERVICES ADMINISTRATION & MANAGEMENT |
| | |



SCHOLARONE™
Manuscripts

BMJ

*I, the Submitting Author has the right to grant and does grant on behalf of all authors of the Work (as defined in the below author licence), an exclusive licence and/or a non-exclusive licence for contributions from authors who are: i) UK Crown employees; ii) where BMJ has agreed a CC-BY licence shall apply, and/or iii) in accordance with the terms applicable for US Federal Government officers or employees acting as part of their official duties; on a worldwide, perpetual, irrevocable, royalty-free basis to BMJ Publishing Group Ltd ("BMJ") its licensees and where the relevant Journal is co-owned by BMJ to the co-owners of the Journal, to publish the Work in this journal and any other BMJ products and to exploit all rights, as set out in our licence.*

*The Submitting Author accepts and understands that any supply made under these terms is made by BMJ to the Submitting Author unless you are acting as an employee on behalf of your employer or a postgraduate student of an affiliated institution which is paying any applicable article publishing charge ("APC") for Open Access articles. Where the Submitting Author wishes to make the Work available on an Open Access basis (and intends to pay the relevant APC), the terms of reuse of such Open Access shall be governed by a Creative Commons licence – details of these licences and which Creative Commons licence will apply to this Work are set out in our licence referred to above.*

*Other than as permitted in any relevant BMJ Author's Self Archiving Policies, I confirm this Work has not been accepted for publication elsewhere, is not being considered for publication elsewhere and does not duplicate material already published. I confirm all authors consent to publication of this Work and authorise the granting of this licence.*

For peer review only

## REVISED MANUSCRIPT- Clean copy 20.02.21

## TITLE PAGE

**Title:** Community health workers' efforts to build health system trust in marginalised communities: A qualitative study from South Africa

**Authors:** Jocelyn Anstey Watkins[1], Frances Griffiths[1,2] and Jane Goudge[2]

**Affiliations**
1. Warwick Medical School, University of Warwick, United Kingdom
2. Centre for Health Policy, School of Public Health, Faculty of Health Sciences, University of the Witwatersrand, Johannesburg, South Africa

**Corresponding author**
Prof Jane Goudge
Email: jane.goudge@gmail.com

**Word count** *(excluding title page, abstract, references, figures and tables/boxes)*: 5625

For peer review only

## ABSTRACT

**Introduction** Community health workers (CHWs) enable marginalised communities, often experiencing structural poverty, to access healthcare. Trust, important in all patient-provider relationships, is difficult to build in such communities, particularly when stigma associated with HIV/AIDS, TB, and now COVID-19, is widespread. CHWs, responsible for bringing people back into care, must repair trust. In South Africa, where a national CHW programme is being rolled out, marginalised communities have high levels of unemployment, domestic violence and injury.

**Objectives** In this complex social environment, we explored CHW workplace trust, interpersonal trust between the patient and CHW, and the institutional trust patients place in the health system.

**Design, Participants, Setting** Within the observation phase of a three-year intervention study, we conducted interviews, focus groups and observations with patients, CHWs, their supervisors and, facility managers in Sedibeng.

**Results** CHWs had low levels of workplace trust. They had recently been on strike demanding better pay, employment conditions and recognition of their work. They did not have the equipment to perform their work safely, and some colleagues did not trust, or value, their contribution. There was considerable interpersonal trust between CHWs and patients, however, CHWs' efforts were hampered by structural poverty, alcohol abuse, and no identification documents among long-term migrants. Those supervisors who understood the extent of the poverty supported CHW efforts to help the community. When patients had withdrawn from care, often due to nurses' insensitive behaviour, the CHWs attempts to repair patient's institutional trust often failed due to the vulnerabilities of the community, and lack of support from the health system.

**Conclusion** Strategies are needed to build workplace trust including supportive supervision for CHWs and better working conditions, and to build interpersonal and institutional trust by ensuring sensitivity to social inequalities and the effects of structural poverty among healthcare providers. Societies need to care for everyone.


## ARTICLE SUMMARY

### Strengths and limitations

- A strength of this research is the number of observational days that the fieldworkers spent with teams of community health workers and other staff across the four sites to understand their daily work and community interactions.
- The resulting rich data illuminates the reality of the vulnerable communities lives in which CHWs work in - desperate poverty, alcoholism, and gender-based violence.
- The combination of data from interviews and observations gave us an in-depth understanding of relationships, and so trust, between the community, community health care workers, their colleagues, supervisors, and in the health system.
- As the data was not collected explicitly for research on trust, participants may not have expressed all their views on trust.
- The study was limited to a peri-urban South African setting, though it is similar to other parts of South Africa and other LMIC contexts.

For peer review only

# MAIN TEXT

## INTRODUCTION

Community health workers (CHWs) serve a critical function in low- and middle-income countries (LMICs), providing frontline services to marginalised groups who face significant barriers to care[1-3]. Effective deployment of CHWs is crucial to moving towards the sustainable development goals (SDG)[4,5]. In settings where HIV and tuberculosis (TB) are established epidemics, CHWs can assist people in adhering to treatment, important where drug resistance to antiretroviral treatment and TB are public health concerns[6,7].

In vulnerable communities, where people experience structural poverty[8], CHWs have to navigate complex health and social situations. Trust, important in all patient-provider-health system relationships[9], is more fragile in such communities[10]. Patients who have unstable lives often receive poorer care; in turn, poor quality care may cause patients to lose trust in their local facility, and become reluctant to seek care in the future[11]. CHWs, responsible for bringing people back into care, must repair that trust, a complex task when stigma associated with HIV/AIDS, TB and now COVID-19[12,13], is widespread.

In South Africa, a national CHW programme is being rolled out, particularly in marginalised communities with high levels of unemployment[14], domestic violence[15] and injury[16], an epidemic fuelled by high rates of alcohol abuse[17]. There is also a high prevalence of communicable and non-communicable conditions[18,19]. Patients struggle to stay in care due to poverty[20] and stigma,[21,22] and those who are migrants face inequity in the health system[23]. CHWs themselves are fighting for employment rights and recognition of their contribution, and so are challenging their relationship with the health system[24]. In this complex social environment, we explored CHW workplace trust, interpersonal trust between the patient and CHW, and the institutional trust patients place in the health system.

## BACKGROUND

### *CHW programme in South Africa*

In 2011, the South African Department of Health, initiated a national CHW programme (ward-based outreach teams; WBOTs) to improve access to services[25]. The intention is to provide health promotion, prevention, screening services and referral for a wide range of health and social needs[26]. The teams are composed of CHWs, supervised by one or two nurses, usually either a retired senior nurse (called a professional nurse) or junior nurse (called an enrolled nurse)[27]. Professional nurses (PNs) in South Africa can diagnose patients, prescribe treatment and dispense medication. PN supervisors are trained in primary healthcare and community nursing. Enrolled nurses (ENs) complete a two-year nursing course and are qualified to provide nursing care under supervision.

CHWs are lay people, members of the community. Prior to the national programme, there were a wide range of CHW programmes managed by a patchwork of NGOs, who in turn were often funded by government, that had emerged in the 1990s due to the HIV epidemic. These CHW were transferred into the government programme as it started in 2011; there was no additional recruitment process. The CHW underwent Phase 1 and 2 standardised training to gain a nationally recognised certificate. This covers identification of the need for ante- and post-natal care, monitoring immunisation and adherence to chronic medication, screening for malnutrition and TB, substance abuse, and gender-based violence[28,29]. CHWs conduct household registrations to identify those in need, and trace patients who have withdrawn from care. During the COVID-19 pandemic, CHWs have been responsible for a mass community-based screening programme, asking people


For peer review only - http://bmjopen.bmj.com/site/about/guidelines.xhtml

about symptoms and referring them to mobile testing units to quarantine suspected cases and provide appropriate care[30].

### *Trust*

Trust, a complex, multifaceted notion, "influences individuals' willingness to act on the basis of words, motives, intentions, actions and decisions of others under conditions of uncertainty, risk or vulnerability"[31]. The existing definitions and theoretical frameworks have done much to elucidate the ambiguity of trust [32]. The social relations of trust are accepted as a core contributor to health systems; a trust-based health system is grounded in cooperation, communication and empathy, enabling the successful functioning of the health service[33]. We have chosen to use the conceptual framework by Gilson et al.[34] which describes the interaction between workplace and patient-provider trust to frame our analysis (see Figure 1). Our purpose is to understand how trust plays out in the workplace of community health workers in South Africa, and from this make recommendations.

*Workplace trust*, defined as respectful and fair treatment in the workplace, is rooted in trust in the employing organisation, trust in your supervisor, and trust in your colleagues. Patient-provider trust is rooted firstly in *interpersonal trust*, in this case between a patient and CHW. Patient-provider trust is also rooted in *institutional trust* - the extent to which the CHW and patient can trust that the health system will support the CHWs to act in the best interests of the patient. Here the health system refers in an immediate sense to the CHW's supervisor, the facility manager, other staff at the facility, as well as the broader health system (access to equipment, medication, effectiveness of referrals to hospital, or social services). Both *interpersonal and institutional trust* are influenced by the individual provider's characteristics, their personalities, past experiences, skills, knowledge, including, for example, sensitivity to patient concerns (e.g. stigma), ability to maintain confidentiality despite living in the same community, and to draw support from the health system. As a result interpersonal trust always influences insitutional level trust.

## METHODS

### Study design

In the initial observation phase of a three-year intervention study in Sedibeng District, Gauteng Province, we studied six CHW teams with different configurations of supervisors and locations. In analysing the observation data (reported elsewhere[35,36]), trust was a re-occurring theme. In this paper, we present the qualitative data from the four, non-intervention teams, to examine the role of trust in greater depth.

### Setting

In Sedibeng, at the time of the study, there were 39 CHW teams in 37 of the district's 72 wards (smallest geopolitical area). Sixteen of the teams were based at a health post and the remaining 23 were clinic-based. A health post consists of one or two temporary wooden structures (providing 3-6 rooms), without electricity and often with irregular water supply. It is managed by one or two PNs. In addition to the scope of practice described above, in Sedibeng the CHWs deliver chronic medication to elderly, or disabled, patients.

The CHWs were not formally employed by the government. They were an outsourced workforce and paid a minimal stipend for six-hours work per day. Two months before the start of our fieldwork, CHWs had been on strike over their conditions of employment. During the fieldwork, the CHWs were paid R2500 (ZAR), below the minimum wage (R3500 per month), however, by the end of 2018, it was increased to the minimum wage. All CHWs were from the local community.



For peer review only - http://bmjopen.bmj.com/site/about/guidelines.xhtml

The four teams served communities that differed by type of population and geography (See Box 1). Housing was either formal brick housing, or informal shacks often made from old corrugated iron, wood and plastic. Residents in the informal settlements are often internal migrants, from poor rural provinces, or long-term migrants, often second generation, originally from Lesotho without South African identification documentation[37], who cannot access government social grants or obtain employment.

**Box 1: Description of the teams and the communities**

**Team 1** – A clinic-based team supervised by a PN and two ENs; well-established, better-off community, with relatively low levels of unemployment. Many residents are elderly to whom the CHWs deliver medication.

**Team 2** – Team based at a health post and supervised by a PN and EN. Largely an informal settlement with very high levels of unemployment, with cases of extreme poverty and child malnutrition.

**Team 3** – Team based at health post supervised by a PN and two ENs; predominately formal brick housing, as well as an area with informal shacks.

**Team 4** – Health post team supervised by a PN and an EN; predominately government-built brick houses with back-room shacks, and a small informal settlement.

## Data collection

Interview and observation data were collected between September 2016 – February 2017 (Table 1). Fieldworkers (FW) were trained in research methods, ethics, and the study tools using extensive role-plays and observation practice. They were also given an orientation to community-based healthcare by an experienced nurse. Interview guides and observation templates (see supplementary material) were revised after piloting and feedback from the fieldworkers[36]. Fieldworkers (none of whom were from the community), introduced themselves to participants as working for the study under the University of the Witwatersrand. Interviews were 15-60 minutes and FGDs 60-90 minutes in duration. No repeat interviews were conducted.

*Observations (obs) of CHWs* - CHWs were selected randomly by drawing names out of a hat, on the first morning of a four-day observation period. The fieldworkers observed CHWs at work in the community, with or without a supervisor. The fieldworkers took detailed notes using a template.

*Focus group discussion* (FGD) topics included descriptions of the types of activities carried out by CHWs and their successes and challenges.

*Key informant interviews* were conducted with the facility manager (FM), and nurse supervisors (EN or PN) to discuss typical activities, resources, how the programme ran, and its successes and challenges.

*Patient interviews* - If during an observational visit a household member was given a referral by a CHW, the fieldworker asked their permission to conduct a follow-up interview one-month later to understand the patient's experience during any follow-up actions.

**Table 1: Qualitative data collected**

| **Field Site** | **Team 1** | **Team 2** | **Team 3** | **Team 4** | **Total** |
|---|---|---|---|---|---|
| **Days of observations with CHWs** | 24 (88) | 18 (43) | 26 (79) | 24 (65) | 92 (275) |


For peer review only - http://bmjopen.bmj.com/site/about/guidelines.xhtml

| | | | | | |
|---|---|---|---|---|---|
| **(number of household visits)** | | | | | |
| **Number of FGDs with CHWs (44 participants)** | 2 | 1 | 2 | 2 | 7 |
| **Number of key informant interviews** | 1 EN<br>1 PN | 1 EN<br>3 PNs<br>1 FM | 2 ENs<br>1 PN<br>1 FM | 1 EN<br>1 PN<br>1 FM | 5 EN<br>6 PN<br>3 FM |
| **Number of patient interviews** | 14 | 6 | 12 | 15 | 47 |

All participants were purposively sampled. All available CHWs participated in the FGDs and all nurse supervisors and facility managers were interviewed. None of the participants were known to the fieldworkers prior to the study. All data collection was conducted in person, audio-recorded and transcribed/translated verbatim by the fieldworkers who wrote reflective notes after each day. Patient interviews were summarised by the fieldworker. We deemed data saturation had been reached when each pair of CHWs had been observed for at least four-days.

## Data analysis

Raw data on trust was extracted from transcripts and notes into a data extraction table for each site by JAW (Research Fellow). Data was placed chronologically so that the stories flowed from day-to-day and we could connect different participant's perspectives on the same issues. Familiarity with the extracted data enabled JAW, JG and FG (all females with PhDs in public health/health science and experienced in qualitative research methods) to develop a common understanding about trust; based on this understanding, JAW identified a range of conceptual frameworks, and we chose Gilson et al.[34] as having the closest fit with the data.

We manually conducted a thematic analysis of the extracted data by drawing out the data on trust, under related themes informed by the framework, into a second data extraction table. [34] We compared data segments and quotes under each theme across the sites and between the participants in an iterative process of reflection. This allowed us to then organise data under each form of trust, as it is presented in the results. We ordered quotes from broad to narrow issues under each theme as this enabled us to develop the logic of our argument.

Participants did not provide feedback on the findings, but one fieldworker was engaged to help check the interpretation of data. We present the results as a narrative including anonymised participant quotations. We draw quotations from across all field sites/teams and across participant groups.

## Ethics

All participants gave informed consent. The project received ethical approval from the University of the Witwatersrand Human Research Ethics Committee (Medical) (M160354), the Gauteng Provincial Health Research Committee, and from the University of Warwick Biomedical and Scientific Research Ethics Sub-Committee (REGO 2016-1825).

## Patient and Public Involvement

CHWs, patients and the public were not involved in the design, conduct, reporting or dissemination plans; however, facility, district and provincial managers were involved. We have conducted feedback sessions with CHW teams, facility, district and provincial managers for the study as a whole.

For peer review only

# RESULTS

## Workplace Trust

### *Conditions of employment*

Many CHWs complained the stipend was not adequate to meet their basic needs and wanted fairer contracts with 21-days of annual leave, and employment after 50-years of age: "We feel that the Department of Health failed us. We were promised that we would be employed permanently, that our stipend would change to a salary." (CHW_FGD2-Team4) During the strike before the fieldwork: "They started toyi-toying [protest dancing], singing the swearing songs at us [staff]. It was just havoc." (FM-Team2) At times, the strike was violent, corroding trust: "The CHWs attacked us and some of the staff were injured." (FM-Team2) Some CHWs continued a strategy of passive resistance: "They did not want to go to the community [to work]; some mentioned that they have nothing to eat at home… one said she will not come to work until she is paid." (FW_obs_CHW-Team2)

### *Working conditions – The health system*

The physical conditions at the health facilities were challenging. At the health post: "The patients wait outside and when it is raining, they get wet. In the consultation rooms, the nurses put buckets because the roof is leaking." (FW_obs_CHW-Team2) At the clinic, the supervisors complained: "We need space to work. It is frustrating because we work in the kitchen." (PN-Team1) The small spaces compromised infection control and made confidentiality difficult. Where there was no space inside, the CHW team met outside, limiting the possibility of discussing patients in confidence.

CHWs work in pairs for safety, even though the CHWs know their communities well: "Sometimes you go to the house with lots of males and we are scared that we might get raped. So, our lives are at risk." (CHW_FGD1-Team4) They were not given adequate personal protective equipment against TB. Other anxieties were not wearing gloves when caring for patients with bedsores, being pricked by a needle, being harmed by dogs, or people living with mental illness.

The CHWs were not supplied with a uniform; their supervisors suggested the CHWs wore black trousers and a white top to make them identifiable and look professional. The CHWs were given work bags containing equipment, however, glucose strips or batteries for the BP monitor were not routinely replaced: "It is so embarrassing when you are at the household, only to find out that the blood pressure machine is not working." (CHW_FGD1-Team3) The CHWs were told that if they should lose the equipment, they would have to replace it. Their bags were routinely searched, and the content was ticked off an inventory by the clinic security guards: "as if they were thieves." (FW_obs _CHW-Team1) One PN threatened to stop their stipend if they did not return their bags each day, even though it was not within her power to do so.

### *Working conditions - The community*

The CHWs are confronted with many complex, and at times, tragic situations: "The child's clinic card was lost. The shack caught fire and the family lost everything." (FW_obs_CHW-Team4) Community members were often intoxicated: "The CHWs find people sitting and drinking a Black Label [beer]. How do you talk to someone who is drinking liquor and talking nonsense?" (PN1-Team2) This made it difficult for the CHWs to do their jobs: "A patient said to us 'I am HIV positive but I don't take antiretrovirals. All I need is men and I survive'. The lady looked drunk. The CHW asked if she drinks alcohol and she said yes, she is trying to reduce stress." (FW_obs_CHW-Team3) The use of drugs was also apparent: "Some households, you find an old woman crying about her misbehaving grandson using nyaope [drugs]. You have to listen and give some counselling and leave when she is feeling better." (FW_obs_CHW-Team1) Descriptions of physical abuse were not uncommon: "The granny started crying. The CHW gave her a tissue to wipe the tears. The granny said the man we passed outside is her husband, who abused her and made her leave home." (FW_obs_CHW-Team1) In another observed visit, when asked how she is doing, a patient replied saying: "she is stressed. She


For peer review only - http://bmjopen.bmj.com/site/about/guidelines.xhtml

showed the CHW her bruises on her arms and said her husband is beating her." (FW_obs_CHW-Team4).

Many community members did not have legal documentation. With no South African identification, children's births are not registered nor are they eligible for government social grants: "The patient does not receive the pension grant because she is from Lesotho. Her son is also unemployed. The CHWs have tried to involve social workers and the police but there is no solution because of the documents." (FW_obs_CHW-Team2) This was a common occurrence, and CHWs are often not able to assist: "Our intervention is not enough because people are not working, those from Lesotho don't have identity documents. You are breaking their hearts because you can't give them anything, besides filling in the registration form and asking: 'Is there someone with TB? Is there someone working?,' when there is no food. You can't even say I will get food parcels, there is nothing." (CHW_FGD-1-Team2) Witnessing such poverty affected the CHWs: "We carry these stories because we are also human. I wish we could have one whole day just to talk about what we have seen and observed." (CHW_FGD-1-Team1)

***Relationships with colleagues***

A hierarchy played out in the health facilities, with the CHWs on the bottom rung of the ladder: "Everyone tells us what to do. We have to do everything we are told to do without any question." (CHW_FGD-1-Team1) CHWs felt their work was not valued: "We have been working here for a long time and no one recognises us. We are just a group of fools." (CHW_FGD-1-Team2) One facility manager (FM) expressed this hierarchy: "The nurses are trained, they know exactly what they are supposed to do, unlike CHWs; they are just called from the streets." (FM-Team2) She justified her distrust: "When we send them out, there are those who will not be doing their work, they will go to their own places to do laundry and clean their houses." (FM-Team2) Her trust had been further eroded during the strike: "It is difficult for us to interact with them because they are very same people who had attacked us. We talk for the purposes of work, other than that, we are keeping them at arm's length." (FM-Team2) Another FM said that despite her grievances about them, "we learn to live with them and realise that these people are useful to us." (FM-Team4)

However, the CHW's supervisors generally praised them. One EN talked of leading the CHWs, even while calling them children: "I know my children [CHWs]. It is important, you must be friendly and polite. Don't be a boss, be a leader. I talk to the CHWs. I tell them 'Be friendly… as I am'." (EN1-Team1) This attitude created a two-way relationship: "The CHWs are comfortable to raise issues with her. If they answer her, 'No sister [nurse], this should be like this and that', she listens to them, and allows them to take initiative and solve the problems." (FW_obs_PN- 1-Team4) Some of the ENs and PNs worked hard to reduce social hierarchy at the clinic, and ensure the CHW abilities were used constructively: "I feel that CHWs are not appreciated. I took my time and studied each CHW. Now I know how to handle each of them. If a CHW is rude, I bring her close to me and tell her that you will be doing statistics with me [collating activity data]." (PN1-Team1) This PN created leadership opportunities: "I said to her [CHW] you are their leader, if they have a problem, they must address it with you [CHW] and you will tell me [PN]." (PN1-Team1) Another PN tried to help the CHWs understand the importance of confidentiality and trusting relationships with patients: "Sometimes, they gossip about their patients and that condemns the family. If they identify a TB patient and I would ask them to adopt that family." This was not always successful "[however] when I ask if they have delivered the treatment to the patient, [the CHW responds] 'why doesn't the patient come and fetch it?'." (PN2-Team2)

Some supervisors viewed the CHWs as "unreliable. They just work for the sake of money… they aren't doing it wholeheartedly." (PN1-Team2) This PN did not trust that they recorded the patient's blood pressure results accurately saying: "[CHWs] are not so honest." (PN1-Team2) One PN would



For peer review only - http://bmjopen.bmj.com/site/about/guidelines.xhtml

belittle the CHWs: “She [PN] even shouts at us in front of the patients. We are adults, mothers and we have our own houses… she does not respect us.” (CHW_FGD-1-Team3)

The less experienced ENs struggled to gain the respect of the CHWs: “I think it is because of my age maybe. They see me as young even though I am a nurse.” (EN1-Team2) The CHWs relied on the PN, dismissing the EN’s help: “The PN is much better, she was able to sit down with us and show us where we went wrong. The EN does not even sit with us. Last time there was a fight here they told her that, ‘you are distant from us’.” (CHW_FGD-1-Team2) The PN was aware this was a problem: “She [EN] has no comradeship with them. I can see that there are some [CHWs] who want to disrespect her because she is still very young.” (PN1-Team2). One EN had little experience, and the CHW had to teach her: “The ENs know nothing about WBOT. We are the ones who had to teach them but they are getting more salary than us. That is not fair; that is why we get angry and strike.” (CHW_FGD-Team4)

However, there was evidence of teamwork between the EN and CHWs: “We have two ENs who are able to walk to the field with us. They are helpful in intervening in our cases.” (CHW_FGD-1-Team1) Another EN was happy to follow the CHWs’ routine, helping when needed: “I don’t change whatever they’re doing that day, because they know their patients. I go and supervise wherever they are going. If they are having any problems, they will take me to those places so that I can help.” (EN1-Team3) The supervisors were key to enlisting the support of other social services, even if their help was limited (Vignette 1).

**Vignette 1**
“I told the police there was a house written, ‘no entry’. The police came and I got into the van and went with them. We found an old lady. There was a small bundle; she was sleeping on some blankets. It was winter. There was bread, a towel, and porridge with nothing else. We bathed her and put on body lotion. After she bathed, she sat in the sun… she said, ‘you remind of the days I used to bathe like this’. I gave her a blanket, towel, underwear, and nappies. I sat down and phoned SASSA [The South African Social Security Agency], and Home Affairs. When they checked her records, she was deported in 1975 back to Lesotho but she found a way to return to South Africa. Home Affairs said there is no way this woman can get an ID book. I am telling you about the problems the WBOT are experiencing.” (PN2-Team2)

## Interpersonal Trust (Patient - CHW)

There was a huge appreciation and respect for the work of the CHWs. “These people found me dying… I was not drinking water, not eating. The CHWs came every morning; they are ever-caring.” (FW_obs_PN-Team4) Relying on the CHWs for emotional support was common: “I have found people that I can pour out my problem to… I feel very good after talking to them.” (Patient-Team3) One man noticed that: “…we [CHWs] sit outside in the sun and he tried to erect a small shack so that we can sit there.” (CHW_FGD-1-Team2) When the CHWs were on strike: “The community elders were even saying: ‘if we were able to walk, we were going to join you [on the strike]’.” (CHW_FGD-1-Team1) Gaining respect, and being acknowledged as a nurse, was hugely motivating: “I feel tall especially when they call me at the [shopping] mall saying ‘Sister!’. Wow, I feel good’.” (CHW_FGD-1-Team1)

Generally, the CHWs were sensitive to people’s personal matters: “We do not tell the mother that her daughter did the HIV test. We can only be free to talk about it if the mother initiates the topic.” (CHW_FGD-2-Team1) Home visits provided the opportunity to build supportive relationships: “I started working with the patient during her pregnancy after she was diagnosed with HIV/AIDS. She was devasted and unfortunately miscarried because of stress and depression. I continued to visit,


For peer review only - http://bmjopen.bmj.com/site/about/guidelines.xhtml

and we developed a good relationship. The patient is taking treatment very well and her CD4 counts have improved." (FW_obs_CHW-Team4) On occasions, CHWs and nurses were not sensitive enough. "The EN asked the teenager if she is sexually active. The girl found the question very difficult to answer in front of her grandmother. One of the CHW's advised the EN to speak with the teenager in a private space. The EN took the advice and used the nearby kitchen to talk with the teenager." (FW_obs_CHW-Team1) The teenager was invited to the health post where she received sex education and contraceptives.

Some community members did not trust the CHWs. The PN supervisor had to reassure a patient that an HIV test would not be done by a CHW. Sometimes people do not answer their doors: "… because they fear that people would think they are HIV positive, but we are visiting everyone." (PN1-Team4) They were blamed for not delivering medications on time: "We go to their houses to deliver medications and they are not there. Then the patient will come to the clinic to complain. So, it seems as if we don't do our work." (CHW_FGD-1-Team1) Occasionally, when the clinic does not have any medication, the CHWs were blamed for selling the medication.

## Institutional Trust (Patient – CHW – Nurse – Health system)

CHWs can make a formal referral to the health facility to obtain care for their patient. In two sites, referred patients did not have to queue at the clinic, but could go straight to the CHW supervisor – this increased the patient's institutional trust of the health system. The teenager's referral (above) enabled a streamlined visit for her first contraception visit: "The referral helped me because it was the first time I visited the clinic. It made it easy as I knew exactly who I was looking for." (PatientTeam1) However, without adequate support from the clinic, CHWs often struggled to support patients: "I had an incident where a patient who tested negative [HIV] throughout her pregnancy but on delivery tested positive. The clinic gave her antiretrovirals but she left them at the clinic. I took them to the woman and she told me, she won't take those them because she is not HIV positive. I had to do counselling which I am not qualified to perform." (CHW_FGD-Team4) The CHW efforts are sometimes frustrated by the clinic 'rules': "Some households do not have food to eat and the patient is on treatment. I contribute something [from my own pocket] so that the patient can eat, but when I ask for porridge from the clinic, the nurse tells me that I have to bring the patient [to the clinic]. That is a big problem because the patient is sick and has no transport money. So, yah it hurts." (CHW_FGD-1-Team3) The nurse was not willing to accept the CHW's word that the person cannot come, and failure to support a patient reduces the CHWs credibility in the community.

In other instances, there was a breakdown in communication with other parts of the system: "She used to go and collect her [government] grant by herself but for the previous two-months she has been bedridden. We promised her a wheelchair. We phoned the hospital, but it was not possible for us to take the old lady to the hospital because of the distance. I don't know what happened [after that]." (FW_obs_CHW-Team2) At times, the complex transitory nature of people's lives made helping them difficult: "A woman was breastfeeding and defaulted on her antiretrovirals, and was refusing to come to the clinic, so they were concerned about the safety of the baby. The CHW referred the patient to the social worker, but she does not think the social worker managed to find the patient because she disappeared." (PN3-Team2)

Some nurses were sensitive to the challenges that the communities faced. "When you sit down and talk with them you would find that they have buckets of stressors. I don't have tablets for stress. I have to try and talk to the person." (PN1-Team2) The same nurse was also aware that her privileged position made her insensitive to patients' challenges: "I asked this woman whether she had bathed or not, because the child was dirty and the woman was also untidy, and she said no. I really got embarrassed because she told me that there was no body soap because there was 'no one working


For peer review only - http://bmjopen.bmj.com/site/about/guidelines.xhtml

at home, we are left with our grandmother, our mother is dead, and we are from Lesotho'." (PN1-Team2)

However, negative attitudes of nurses often affected the patient's willingness to attend the health facility: "The CHWs asked an old lady why she is not taking her treatment. The lady said the sister [nurse] at the clinic doesn't talk to her well, so it is better for her to stop going to the clinic." (FW_obs_CHW-Team3) Some patients confided in the CHWs about the nurses' behaviour: "[the patient] said if the nurse comes to their houses and speaks the way she speaks when she is at the clinic, they are going to hit her." (CHW_FGD-2-Team3) Vignette 2, describes a woman who was no longer taking her antiretroviral treatment due to a disagreement with the health post staff. In vignette 3, a nurse is rude to a vulnerable pregnant woman who withdraws from care; the CHW enlists the PN's support to get the nurse to apologise but fails.

**Vignette 2**

The CHW asked the woman why she is breastfeeding whilst she is HIV positive and not taking treatment: "She said she and her child are both fine, the child has never been sick, and she is picking up weight: 'I will see about that thing when I am sick'." (FW_obs_CHW-Team4) The nurse had taken bloods from her baby, but the results were lost. Before retesting the child, the woman was insisting: "The clinic must first tell me what happened to the blood that was drawn." (FW_obs_CHW-Team4)

**Vignette 3**

A patient was raped as a teenager and contracted HIV. Her first child passed away and her second child was also HIV positive. The patient usually takes antiretrovirals. She is pregnant. When visiting the clinic, a nurse refused to check the unborn baby because the mother did not have a transfer letter from her previous clinic in the Eastern Cape. The consultation ended with the nurse saying: "I don't care if you give birth in the toilet or in the street. It is not my problem." (FW_obs_CHW-Team3) Feeling angry and upset, the pregnant woman refused to go back to the clinic. With tears flowing down her face the woman said: "I know the rules of the clinic for a pregnant mother who is HIV positive, and I follow them always. I did not choose to be HIV positive." (Patient-Team3) The CHW promised to collect her medication on her behalf, and also she would report the case to the supervisor, saying: "she [patient] must not worry as she is not the first one she has treated badly and this time she [CHW] is going to report her." (FW_obs_CHW-Team3) However after the patient and nurse communicated, the patient reported: "the sister [nurse] denied all the things she said to me, they wrote on my clinic card that I will never come back to this clinic, and the clinic will not be accountable should anything happen to me." (Patient-Team3)

Responsible for finding defaulting patients, the CHWs have to find ways to repair the patient's trust in the health system: "We talk to the patients, encourage them to go for the sake of their health and ignore the nurses' attitude." (CHW_FGD-2-Team3) However, the CHWs could not guarantee patients will be treated better next time: "I [CHW] told the patient that when you [don't go to] the clinic, you are killing yourself as you are the one who is taking treatment, not the sister [nurse] and the life that you are living is yours but not the sister's." (CHW_FGD-1-Team3) A few people said they would only return to seek care if the CHW accompanied them: "Okay, I will come only if you [CHW] will be there too." (FW_obs_CHW-Team4)

## DISCUSSION

In this paper, we have explored both workplace trust, patient-CHW interpersonal trust and patient-health system (institutional) trust, amongst CHW teams in South Africa. The CHWs are the lowest



For peer review only - http://bmjopen.bmj.com/site/about/guidelines.xhtml

cadre in the health system; their conditions of employment, their working environment, the lack of necessary equipment to perform their work safely, and the treatment by some colleagues indicated to the CHWs that their work was unrecognised, their contribution untrusted. The low levels of monetary incentives and poor working conditions of CHWs have been reported elsewhere in South Africa[38], and other resourced constrained settings[39]. By striking, the CHWs in our study were demanding better pay and employment conditions, as well as recognition of the importance of their work.

The CHWs are working in communities mired in complex social problems the result of long-term structural poverty. Dysfunctional family relationships impact on patients' ability to look after themselves and take their treatment. Alcohol abuse blights the lives of community members, making the work of the CHWs harder. The lack of identification documents among long-term migrants leads to desperate poverty that makes accessing healthcare difficult, even with the CHWs' efforts. Some supervisors understood the extent of the poverty in the surrounding communities, from which the CHWs themselves came. These supervisors worked hard to overcome the social hierarchy by building the CHW's skills, and supporting their efforts to help the community.

There was considerable evidence of interpersonal trust between patients and CHWs, with many people appreciative of their work. CHW's operate in a unique environment, where household visits enable strong relationships to be built, but living in the same community can test the CHWs' ability to maintain confidentiality[10]. CHWs have to make sensitive judgements about when and what to ask people in order to build trust, a difficult terrain to navigate, particularly because of the vulnerability of many of their patients.

The attitude of some of the facility-based nurses ("bad apples"[40]) led some patients to withdraw from care[41]. Insensitive to stigma and barriers to accessing care that the socio-economic conditions of people's lives create, nurses' behaviour offended patients. CHW's attempts to repair trust often failed due to the vulnerabilities of the community, and lack of support from the health system, underpinned by poor workplace trust including CHWs often fraught relationships with their colleagues. We have reported elsewhere that inadequate and unpredictable support from the clinic negatively affects the CHW's ability to provide care and in turn, their credibility in the community[35].

Migrants, often unable to seek care because of their poverty, are being denied a fundamental human right. Section 27 of the 1996 South African Constitution states that "everyone has the right to have access to health services". The National Health Insurance (NHI) Bill[42] states that illegal and undocumented migrants "will receive basic healthcare services" (emergency care and treatment for HIV, TB and malaria), but not general primary healthcare or sexual and reproductive services[43]. It is not possible to provide effective HIV treatment without related primary healthcare services[44], and this is at odds with the SDG of 'leaving no one behind'[45] and achieving universal health coverage. Societies need to care for everyone in them.

### Strengths and limitations

A strength of this research is the number of observational days that the fieldworkers spent with the community health workers and other staff across the four sites to understand their daily work and community interactions. The resulting rich data illuminates the reality of the vulnerable communities lives in which CHWs work in - desperate poverty, alcoholism, and gender-based violence, as well as the relationships, and so trust, between the community, community health care workers, their colleagues, supervisors, and in the health system. As the data was not collected explicitly for research on trust, participants may not have expressed all their views on trust. Moreover, text describing relationships, based on individual perceptions, is often difficult to



For peer review only - http://bmjopen.bmj.com/site/about/guidelines.xhtml

interpret – we suggest that our detailed knowledge of the context, and our rigorous analysis process described above, has ensured that our interpretations are true to our participants' experiences.

## Recommendations

Given the interconnected nature of workplace, interpersonal and institutional trust, our recommendations include:

1. CHWs and nurses should be provided with opportunities to develop a better understanding of, and empathy for, the community's health and social situation.
2. Facility managers and nurses need to work to overcome social hierarchy in the facility, so CHWs feel supported in their workplace and patients feel cared for.
3. Inexperienced ENs need to be mentored while they develop as CHW supervisors.
4. In communities with complex social problems, the CHWs and their supervisors need strong intersectoral collaborations with other services.
5. Migrants need to have the right, and means, to be able to access care.

## Conclusion

CHWs' role in enabling vulnerable communities to access care is underpinned by workplace, interpersonal trust and institutional trust. Without these different forms of trust, CHWs struggle to assist patients to stay in care; yet creating trust in marginalised communities struggling with structural poverty is far from easy. Nurses and CHW supervisors need to be sensitive to the hierarchy created by social inequalities and the barriers that patients face in accessing care. They need to support the CHWs in helping patients overcome these barriers. The government's role in ensuring migrants' rights to accessing healthcare services is crucial in developing trust.

## Acknowledgements

The authors would like to thank the Sedibeng community and primary healthcare staff for being involved in the study. Thank you, also to the dedicated team of fieldworkers working for the Bathlokomedi project.

## Contributionship

JG and FG are the principal investigators and award holders on this grant, conceptualising the study and managing data collection. They contributed to the supervision, data analysis and interpretation and drafting this article. JAW extracted and analysed the data and wrote the article, with JG editing drafts. All authors reviewed the final draft of this article.

## Funding

The work was supported by the UK Medical Research Council [grant number: MR/N015908/1].

## Competing Interests

The authors declare that they have no financial or personal relationships that may have inappropriately influenced them in writing this article.

## Patient Consent for publication

Obtained.

## Data sharing statement

For peer review only

Data is available upon request from Prof Jane Goudge (jane.goudge@gmail.com).

**Figure 1. Conceptual framework on trust adapted from Gilson et al., 2005**

## REFERENCES


1. Kok MC, Ormel H, Broerse JE, et al. Optimising the benefits of community health workers' unique position between communities and the health sector: a comparative analysis of factors shaping relationships in four countries. *Global Public Health* 2017;12(11):1404-32. doi: 10.1080/17441692.2016.1174722
2. Daniels K, Odendaal WA, Nkonki L, et al. Incentives for lay health workers to improve recruitment, retention in service and performance. *The Cochrane Database of Systematic Reviews* 2019;2019(12) doi: 10.1002/14651858.CD011201
3. Ludwick T, Morgan A, Kane S, et al. The distinctive roles of urban community health workers in low-and middle-income countries: a scoping review of the literature. *Health Policy and Planning* 2020;czaa049 doi: doi.org/10.1093/heapol/czaa049
4. Wahl B, Lehtimaki S, Germann S, et al. Expanding the use of community health workers in urban settings: a potential strategy for progress towards universal health coverage. *Health Policy and Planning* 2020;35(1):91-101. doi: 10.1093/heapol/czz133
5. Maher D, Cometto G. Research on community-based health workers is needed to achieve the sustainable development goals. World Health Organisation. Geneva. 2016 [Available from: https://www.who.int/bulletin/volumes/94/11/16-185918/en/ accessed 29th July 2020.
6. Chimukangara B, Lessells RJ, Rhee S-Y, et al. Trends in pretreatment HIV-1 drug resistance in antiretroviral therapy-naive adults in South Africa, 2000–2016: a pooled sequence analysis. *EClinicalMedicine* 2019;9:26-34. doi: 10.1016/j.eclinm.2019.03.006
7. Streicher EM, Müller B, Chihota V, et al. Emergence and treatment of multidrug resistant (MDR) and extensively drug-resistant (XDR) tuberculosis in South Africa. *Infection, Genetics and Evolution* 2012;12(4):686-94. doi: 10.1016/j.meegid.2011.07.019
8. Rylko-Bauer B, Farmer P. Structural violence, poverty, and social suffering. *The Oxford Handbook of the Social Science of Poverty* 2016:47-74.
9. Brennan N, Barnes R, Calnan M, et al. Trust in the health-care provider–patient relationship: a systematic mapping review of the evidence base. *International Journal for Quality in Health Care* 2013;25(6):682-88. doi: 10.1093/intqhc/mzt063
10. Grant M, Wilford A, Haskins L, et al. Trust of community health workers influences the acceptance of community-based maternal and child health services. *African Journal of Primary Health Care & Family Medicine* 2017;9(1):1-8. doi: 10.4102/phcfm.v9i1.1281
11. Eyles J, Harris B, Fried J, et al. Suspicious minds: Apportioning and avoiding blame for distrustful relationships and deferring medical treatment in South Africa. *Sociology Mind* 2015;5(03):188. doi: 10.4236/sm.2015.53017
12. Besada D, Daviaud E. If we invested in this today, South Africa could save billions — and fight COVID-19. Bhekisisa Centre for Health Journalism. South Africa. 2020 [1st July 2020]. Available from: https://bhekisisa.org/opinion/2020-05-20-community-health-care-workers-in-south-africa-investment-case-covid19-coronavirus-tracing-programme/ accessed 1st July 2020.
13. Xolo N. Concerns mount over Covid-19 stigma in KZN. Maverick Citizen South Africa, 2020 [Available from: https://www.dailymaverick.co.za/article/2020-05-25-concerns-mount-over-covid-19-stigma-in-kzn/#gsc.tab=0 accessed 22nd July 2020.


For peer review only



For peer review only - http://bmjopen.bmj.com/site/about/guidelines.xhtml

14. Fransman T, Yu D. Multidimensional poverty in South Africa in 2001–16. *Development Southern Africa* 2019;36(1):50-79. doi: 10.1080/0376835X.2018.1469971
15. Meyiwa T, Williamson C, Maseti T, et al. A twenty-year review of policy landscape for gender-based violence in South Africa. *Gender and Behaviour* 2017;15(2):8607-17.
16. Seedat M, Van Niekerk A, Jewkes R, et al. Violence and injuries in South Africa: prioritising an agenda for prevention. *The Lancet* 2009;374(9694):1011-22. doi: 10.1016/S0140-6736(09)60948-X
17. Herrick C, Parnell S. Alcohol, poverty and the South African city. *South African Geographical Journal* 2014;96(1):1-14. doi: 10.1080/03736245.2014.896277
18. Oni T, Youngblood E, Boulle A, et al. Patterns of HIV, TB, and non-communicable disease multi-morbidity in peri-urban South Africa-a cross sectional study. *BMC Infectious Diseases* 2015;15(1):1-8. doi: 10.1186/s12879-015-0750-1
19. Mayosi BM, Flisher AJ, Lalloo UG, et al. The burden of non-communicable diseases in South Africa. *The Lancet* 2009;374(9693):934-47. doi: 10.1016/S0140-6736(09)61087-4
20. Goudge J, Gilson L, Russell S, et al. Affordability, availability and acceptability barriers to health care for the chronically ill: longitudinal case studies from South Africa. *BMC Health Serv Res* 2009;9:75. doi: 10.1186/1472-6963-9-75
21. Kalichman SC, Mathews C, Banas E, et al. Treatment adherence in HIV stigmatized environments in South Africa: stigma avoidance and medication management. *International Journal of STD & AIDS* 2019;30(4):362-70. doi: 10.1177/0956462418813047
22. Mabunda K, Ngamasana EL, Babalola JO, et al. Determinants of poor adherence to antiretroviral treatment using a combined effect of age and education among human immunodeficiency virus infected young adults attending care at Letaba Hospital HIV Clinic, Limpopo Province, South Africa. *The Pan African Medical Journal* 2019;32 doi: 10.11604/pamj.2019.32.37.17722
23. Vearey J, Modisenyane M, Hunter-Adams J. Towards a migration-aware health system in South Africa: A strategic opportunity to address health inequity. *South African Health Review* 2017;2017(1):89-98.
24. Trafford Z, Swartz A, Colvin CJ. "Contract to Volunteer": South African community health worker mobilization for better labor protection. *New Solutions: A Journal of Environmental and Occupational Health Policy* 2018;27(4):648-66. doi: 10.1177/1048291117739529
25. SADoH. Provincial guidelines for the implementation of the three streams of PHC Re-engineering: South African Department of Health; 2011 [Available from: http://www.jphcf.co.za/wp-content/uploads/2014/06/GUIDELINES-FOR-THE-IMPLEMENTATION-OF-THE-THREE-STREAMS-OF-PHC-4-Sept-2.pdf accessed 19th August 2020.
26. Schneider H, Schaay N, Dudley L, et al. The challenges of reshaping disease specific and care oriented community based services towards comprehensive goals: a situation appraisal in the Western Cape Province, South Africa. *BMC Health Services Research* 2015;15(1):436. doi: 10.1186/s12913-015-1109-4
27. Mhlongo EM, Lutge E. The roles, responsibilities and perceptions of community health workers and ward-based primary health care outreach teams (WBPHCOTs) in South Africa: a scoping review protocol. *Systematic Reviews* 2019;8(1):1-7. doi: 10.1186/s13643-019-1114-5
28. Griffiths F, Babalola O, Brown C, et al. Development of a tool for assessing quality of comprehensive care provided by community health workers in a community-based care programme in South Africa. *BMJ Open* 2019;9(9):e030677. doi: 10.1136/bmjopen-2019-030677
29. SAQA. National Certificate: Community Health Work Phase 1 and 2. South African Qualifications Authority 2018 [Available from: https://qspe.saqa.org.za/showQualification.php?id=64749 accessed 10th August 2020.


For peer review only - http://bmjopen.bmj.com/site/about/guidelines.xhtml

30. Daniel L. Coronavirus: what is South Africa COVID home-visits-program South Africa, 2020 [Available from: https://www.thesouthafrican.com/news/coronavirus-what-is-south-africa-covid-home-visits-program/ accessed 22nd July 2020.
31. Okello DR, Gilson L. Exploring the influence of trust relationships on motivation in the health sector: a systematic review. *Human Resources for Health* 2015;13(1):16. doi: 10.1186/s12960-015-0007-5
32. Gopichandran V, Chetlapalli SK. Dimensions and determinants of trust in health care in resource poor settings–a qualitative exploration. *PLoS One* 2013;8(7):e69170.
33. Gilson L. Trust and the development of health care as a social institution. *Social Science & Medicine* 2003;56(7):1453-68. doi: 10.1016/S0277-9536(02)00142-9
34. Gilson L, Palmer N, Schneider H. Trust and health worker performance: exploring a conceptual framework using South African evidence. *Social Science & Medicine* 2005;61(7):1418-29. doi: 10.1016/j.socscimed.2004.11.062
35. Tseng Y-h, Griffiths F, de Kadt J, et al. Integrating community health workers into the formal health system to improve performance: a qualitative study on the role of on-site supervision in the South African programme. *BMJ Open* 2019;9(2):e022186. doi: 10.1136/bmjopen-2018-022186
36. Goudge J, Kadt Jd, Babalola O, et al. Household coverage, quality and costs of care provided by community health worker teams and the determining factors: Findings from a mixed methods study in South Africa. *BMJ Open* 2020;10:e035578. doi: 10.1136/bmjopen-2019-035578
37. Segatti A, Landau L. Contemporary migration to South Africa: a regional development issue: The World Bank 2011.
38. Maboko S, Hlongwana K, Mashamba-Thompson TP. Factors influencing the motivation of community health workers in Vhembe district, Limpopo: case study. *South African Health Review* 2018;2018(1):67-68.
39. Singh D, Negin J, Otim M, et al. The effect of payment and incentives on motivation and focus of community health workers: five case studies from low-and middle-income countries. *Human Resources for Health* 2015;13(1):1-12. doi: 10.1186/s12960-015-0051-1
40. Kane S, Calnan M, Radkar A. Trust and trust relations from the providers' perspective: the case of the healthcare system in India. *Indian Journal of Medical Ethics* 2015;12(3):157-68. doi: 10.20529/IJME.2015.045
41. Nkosi B, Seeley J, Ngwenya N, et al. Exploring adolescents and young people's candidacy for utilising health services in a rural district, South Africa. *BMC Health Services Research* 2019;19(1):195. doi: 10.1186/s12913-019-3960-1
42. NDOH. South African National Health Insurance White Policy Paper. *The National Department of Health, South Africa* 2017
43. Section27. Spotlight on NHI: NHI Bill will divert SA from Universal Health Coverage if some groups are excluded. South Africa, 2019 [Available from: http://section27.org.za/2019/11/spotlight-on-nhi-nhi-bill-will-divert-sa-from-universal-health-coverage-if-some-groups-are-excluded/ accessed 6th August 2020.
44. Faturiyele I, Karletsos D, Ntene-Sealiete K, et al. Access to HIV care and treatment for migrants between Lesotho and South Africa: a mixed methods study. *BMC Public Health* 2018;18(1):668. doi: 10.1186/s12889-018-5594-3
45. Stuart E, Woodroffe J. Leaving no-one behind: can the Sustainable Development Goals succeed where the Millennium Development Goals lacked? *Gender & Development* 2016;24(1):69-81. doi: 10.1080/13552074.2016.1142206


For peer review only

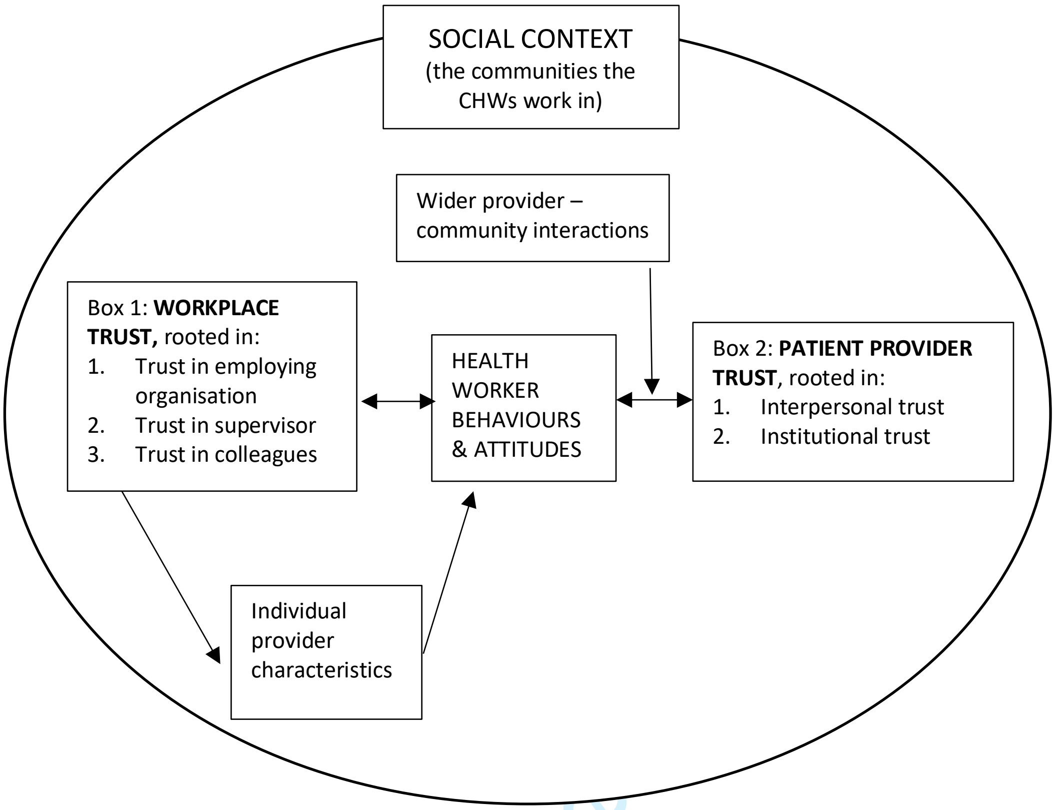


For peer review only

V0.95; 12 September 2016

# Interview Guides for the Bathlokomedi project

**Annex A-G**

**Annex A: Information Sheet for interviewing the facility manager of the 'mother' clinic.**

**Annex B: Information Sheet for interviewing Nurse team leader of the CHW team and Field version Nurse team leader of CHW interviews**

**Annex C: Information Sheet for observing community health workers**

**Annex D: Information Sheet for observing nurse leader of community health worker team**

**Annex E: Information sheet for group interview with community health workers**

**Annex G: Information Sheet for interviewing referred householder**

## Annex A: Information Sheet for interviewing the facility manager of the 'mother' clinic.

**Formal Title: Implementing comprehensive, integrated, community-based health care for under-served, vulnerable communities in South Africa: A practical, evidence-informed model.**

Hello, and thank you for your time today.

My name is ________. I work for the Centre for Health Policy at the University of the Witwatersrand. I am a researcher in the Batlhokomedi project, and I would like to interview you today as part of this project.

What is this research about?

We are conducting this research to understand how community health worker / WBOT programmes operate. We know that there are some innovative WBOT programmes in Sedibeng and we are keen to learn more about your programmes here. I believe you are the manager of this clinic that has some WBOT teams. So we would like to hear about your experiences of having WBOTs associated with your clinic, both the successes and challenges.

The interview should take about 40 mins.

Voluntary Participation

It is up to you to decide whether you want participate in this study. If you agree to help with this research, and later change your mind, you are free to end the interview at any time, without any consequences.

Risks

There are no risks associated with talking to me today.



For peer review only - http://bmjopen.bmj.com/site/about/guidelines.xhtml

V0.95; 12 September 2016

Confidentiality

No one other than my team members will be allowed to see the record of the interview discussion. We will not use your name in any reports of this work. We will use a code instead of your name e.g. A or B.

My notes and audio recordings will be kept on a secure university computer server, and participant codes will be kept in a locked filing cabinet under the care of the project site manager, and will be destroyed once our research is complete.

Approval for and benefits of this work

The study has been approved by the ethics committee of University of the Witwatersrand, and Gauteng Province. We hope the study will contribute new ideas and insights regarding the provision of community health worker services.

If you agree to be interviewed, we would also like your permission to record the interview as it really helps later in the office.

What if I have any questions?

You are free to ask me any question about this research. If you have any further questions about the study, you are free to contact Julia De Kadt on Julia.DeKadt2@wits.ac.za, or 011-717-3434 or 074 336 9411.
If you are concerned about anything to do with the study in general, or wish to make a complaint, you can contact the chair of the ethics committee at the University of the Witwatersrand, who is Professor Peter Cleaton-Jones at telephone number: (011) 717-2301.

## Tool A: Interview managers of the 'mother' clinic to which the CHW team

**Introduction**

Thank you again for agreeing to be interviewed. Are you happy to start?

**First, I want to ask you some questions about yourself. Please tell me a bit about yourself?**

Probes:
How many years have you worked as a nurse?
What is your professional title? (eg. Enrolled nurse, Professional nurse, other specialized nurse)
How many years have you worked at this facility?
How many years in the current position?
Were you born in this district?

**Perhaps we could talk now about the relationship between the clinic and the CHW team. Please can you describe the relationship between the 'mother' clinic and the CHW team?**

Probes:
How long has the CHW team been working from / linked to this clinic?
What role does the clinic play in the recruitment of CHWs?
What role the 'mother' clinic has in supporting the CHW team?
What happens when patients are referred here by CHW? Who do they see?
How does the data reporting system work?
What else could the CHWs do to better support the clinic?

**What is your role as a clinic manager with respect to the CHW team?**



For peer review only - http://bmjopen.bmj.com/site/about/guidelines.xhtml

V0.95; 12 September 2016

Probes:
***Where team leader nurse is based in clinic***
How much time do you expect the team leader to spend managing the CHW team?
Can you describe how she manages to balance her time between her clinic duties and the CHW team?
What happens when the team leader is not here?

***Both where the team leader is based at the health post and the clinic***
What engagement happens between you and CHW team leader? Between the CHW team and the clinic staff? What sort of things do they communicate about?

**What have you been able to achieve with CHW?**

Probes:
What are the successes the CHW team have been able to achieve?

**What are the challenges?**

Probes:
Have you ever had a complaint from the community about a CHW? What happened?

**How do you engage with the community?**

Probes:
What do the community think of the community health workers?
How does the community express their views?
Are there other organisations providing health care in the community and what do they do?
What is the role of the clinic committee? How effective is it?
Are training and tasks of the CHWs in line with local needs?

**Ending the interview**
Thank you. I think we've covered all of the questions that I wanted to ask you today. Do you have anything else you wanted to add?

**Topics we would like to hear about from the interviewees:**
recent health campaigns and role of community health workers;
access to supplies, transport, advice, equipment, space to meet and store;
referral management and feedback;
language barriers for community health workers; safety and security of CHW in the community;
previous training and experience of CHW;
other duties of nurse/manager;
incentives for the CHW;
complaints against a CHW and what happened.

**Suggested generic prompts to use to encourage participants to continue:**

*To get going on a topic if they are unsure of where to start or what to say:*
Start wherever you want
Start with what you think is most important
Why not start by telling me what happens in a day, starting at the beginning

*To go into more depth or to encourage continuation of their account*

For peer review only

V0.95; 12 September 2016

Please tell me more about that
Please explain that for me
Why was that?
What did you feel about that?
What happened then?
Please can you tell me more about (person, role, place, organisation, activity etc.)

## Annex B: Information Sheet for interviewing Nurse team leader of the CHW team

**Formal Title: Implementing comprehensive, integrated, community-based health care for under-served, vulnerable communities in South Africa: A practical, evidence-informed model.**

Hello, and thank you for your time today.

My name is ________. I work for the Centre for Health Policy at the University of the Witwatersrand. I am a researcher in the Batlhokomedi project, and I would like to interview you today as part of this project.

<u>What is this research about?</u>
We are conducting this research to understand how community health worker / WBOT programmes operate. We know that there are some innovative WBOT programmes in Sedibeng and we are keen to learn more about your programmes here. I believe you are the leader of a WBOT team. So we would like to hear about your experiences of leading a team of CHWs, both the successes and challenges.

The interview should take about 40 mins.

<u>Voluntary Participation</u>
It is up to you to decide whether you want participate in this study. If you agree to help with this research, and later change your mind, you are free to end the interview at any time, without any consequences.

<u>Risks</u>
There are no risks associated with talking to me today.

<u>Confidentiality</u>
**<u>No one other than my team members will be allowed to see the record of the interview discussion. We will not use your name in any reports of this work. We will use a code instead of your name e.g. A or B.</u>**

My notes and audio recordings will be kept on a secure university computer server. Participant codes will be kept in a locked filing cabinet under the care of the project site manager, and will be destroyed once our research is complete.

<u>Approval for and benefits of this work</u>
The study has been approved by the ethics committee of University of the Witwatersrand, and Gauteng Province. We hope the study will contribute new ideas and insights regarding the provision of community health worker services.

If you agree to be interviewed, we would also like your permission to record the interview as it really helps later in the office.


For peer review only - http://bmjopen.bmj.com/site/about/guidelines.xhtml

V0.95; 12 September 2016

What if I have any questions?
You are free to ask me any question about this research. If you have any further questions about the study, you are free to contact Julia De Kadt on Julia.DeKadt2@wits.ac.za, or 011-717-3434 or 074 336 9411.

If you are concerned about anything to do with the study in general, or wish to make a complaint, you can contact the chair of the ethics committee at the University of the Witwatersrand, who is Professor Peter Cleaton-Jones at telephone number: (011) 717-2301

## Annex B Field version Nurse team leader of CHW interviews

**Introduction**

Thank you again for agreeing to be interviewed. Are you happy to start?

**First, could you please tell me a bit about yourself?**
Probes:
How many years have you worked as a nurse?
What is your professional title? (eg: Enrolled nurse, Professional nurse, other specialized nurse)
How many years have you worked at this facility?
How many years in the current position?
Were you born in this district?

**Perhaps we could talk now about the Ward Based Outreach Team. Please tell me about how the Ward Based Outreach Team is run?**
Probes:
How are community health workers recruited to the team?
Are there different types of CHW, with different training?
What resources are provided to CHW?
What happens in a typical day of the team?
How is the team's work for the day planned?
What happens on different days in the week?
How often do CHWs work after hours on weekends, and what do they do then?
How is work distributed within the team? Is there any division of labour? Do any CHWs have a leadership role within the team?
How does the data reporting system work?

- **What information do CHWs record while out in the community?**
- **What is done with this information? When?**
- **What forms are used?**
- **What is your role in the data reporting system? What exactly do you do?**

How does the referral system work?
Are there language barriers for WBOT?
Are there safety and security issues when working in the community? If so, what precautions do you take to ensure the safety of the CHW? What incidents are you aware of happening to the WBOT?
What incentives are there for the WBOT?

*We would expect the following activities to be mentioned: advice giving to households; registration of households, case finding; referral management and feedback; recent health campaigns and role of community health workers.*

**What are your roles and responsibilities for WBOT?**

*Probes:*



For peer review only - http://bmjopen.bmj.com/site/about/guidelines.xhtml

V0.95; 12 September 2016

What happens in a typical day for you?
What happens on different days in the week?
What happens when you are away?
What other responsibilities do you have at work, other than WBOT? Roughly, how much of your time do those activities take?
Please tell me about times when you have faced conflicting demands?
What engagement happens between you and manager and other staff at the clinic?
What engagement happens between the CHW team and the clinic staff?
What support do you receive for your work from colleagues ?

- **From District management**

What resources – financial, equipment, supplies, place to work, place to meet and store, transport, airtime

Do you receive any support from the community ?

What additional support would be helpful?

What dilemmas do you face in your day to day work?

**What have you been able to achieve with WBOT?**
*Probes:*
What are you proud of with respect to the achievements of WBOT?
What do you think are the key factors in achieving this?
What would enable you to achieve more?

**What are the challenges?**
Probes:
What challenges do you experience?
What do you do if one of the CHW is not doing their job very well?
Have there been any complaints against WBOT? What was it about? What happened?
Can you tell us anything about the strike earlier this year?

- **What happened?**
- **Have the issues that caused the strike been addressed?**

**How do you engage with the community?**
*Probes:*
What do the community think of the community health workers?
How does the community express their views?
Are training and services of CHWs well matched to local needs?
Can you tell me about the clinic committee? What sort of things do they engage with you about ?
Are there other organisations providing health care in the community? What do they do?
How do you work with these organisations?

**Ending the interview**
Thank you. I think we've covered all of the questions that I wanted to ask you today. Do you have anything else you wanted to add?

**Suggested generic prompts to use to encourage participants to continue:**
*To get going on a topic if they are unsure of where to start or what to say:*
Start wherever you want
Start with what you think is most important


For peer review only - http://bmjopen.bmj.com/site/about/guidelines.xhtml

Why not start by telling me what happens in a day, starting at the beginning

*To go into more depth or to encourage continuation of their account*
Please tell me more about that
Please explain that for me
Why was that?
What did you feel about that?
What happened then?
Please can you tell me more about (person, role, place, organisation, activity etc.)

## Annex C: Information Sheet for observing community health workers

**Formal Title:** Implementing comprehensive, integrated, community-based health care for under-served, vulnerable communities in South Africa: A practical, evidence-informed model.

Hello and thank you for your time today. My name is ________. I work for the Centre for Health Policy at the University of the Witwatersrand. I am a researcher in the Batlhokomedi project, and I would like to observe you today as part of this project.

**What is this research about?**
We are conducting this research to understand how community health worker / WBOT programmes operate. We know that there are some innovative WBOT programmes in Sedibeng and we are keen to learn more about your programmes here. I know you are a community health worker, so I would like to observe what you do as a community health worker. Without disrupting your work, I would also like to ask you about what you are doing while we are observing.

If you agree, while we are observing you and talking to you, we will take notes and then type these notes up.

Voluntary Participation
It is up to you to decide whether you want participate in this study. If you agree to help with this research, and later change your mind, you are free to withdraw at any time, without any consequences.

Risks
There are no risks associated with participating in the research.

Confidentiality
The information will be used for research purposes only. No one other than my team members will be allowed to see the record of the observation. We will not use your name in any reports of this work. We will use a code instead of your name e.g. A or B.

My notes will be kept on a secure university computer server, and participant codes will be kept in a locked filing cabinet under the care of the project site manager, and will be destroyed once our research is complete.

Approval for and benefits of this work



For peer review only - http://bmjopen.bmj.com/site/about/guidelines.xhtml

V0.95; 12 September 2016

The study has been approved by the ethics committee of University of the Witwatersrand, and Gauteng Province. The study will contribute new ideas and insights regarding the provision of community health worker services.

What if I have any questions?

You are free to ask me any question about this research. If you have any further questions about the study, you are free to contact Julia de Kadt on julia.dekadt2@wits.ac.za or 011 717 3434 or 074 336 9411.

If you are concerned about anything to do with the study in general, or wish to make a complaint, you can contact the chair of the ethics committee at the University of the Witwatersrand, who is Professor Peter Cleaton-Jones at telephone number: (011) 717-2301

## Annex D: Information Sheet for observing nurse leader of community health worker team

**Formal Title: Implementing comprehensive, integrated, community-based health care for under-served, vulnerable communities in South Africa: A practical, evidence-informed model.**

Hello, and thank you for your time today. My name is ________. I work for the Centre for Health Policy at the University of the Witwatersrand. I am a researcher in the Batlhokomedi project, and I would like to observe you today as part of this project.

**What is this research about?**

We are conducting this research to understand how community health worker / WBOT programmes operate. We know that there are some innovative WBOT programmes in Sedibeng and we are keen to learn more about your programmes here. I believe you are the leader of a WBOT team. So we would like to observe what you do as a nurse, and what activities you do to support the CHWs. Without disrupting your work, we would also to ask you about what you are doing while we are observing. We do not wish to observe any consultations with patients in the clinic or health post, although we wish to observe home visits if you conduct any.

If you agree, while we are observing you and talking to you, we will take notes and then later type these notes up.

Voluntary Participation

Participation in this study is voluntary. If you agree to help with this research and later change your mind you are free to withdraw at any time, without any consequences.

Risks

There are no risks associated with participating in the research.

Confidentiality



For peer review only - http://bmjopen.bmj.com/site/about/guidelines.xhtml

V0.95; 12 September 2016

The information will be used for research purposes only. No one other than my team members will be allowed to see the record of the observation. We will not use your name in any reports of this work. We will use a code instead of your name e.g. A or B.

My notes will be kept on a secure university computer server. Participant codes will be kept in a locked filing cabinet under the care of the project site manager, and will be destroyed once our research is complete.

Approval for and benefits of this work

The study has been approved by the ethics committee of University of the Witwatersrand, and Gauteng Province. The study will contribute new ideas and insights regarding the provision of community health worker services.

What if I have any questions?

You are free to ask me any question about this research. If you have any further questions about the study, you are free to contact Julia de Kadt on julia.dekadt2@wits.ac.za or 011 717 3434 or 074 336 9411.

If you are concerned about anything to do with the study in general, or wish to make a complaint, you can contact the chair of the ethics committee at the University of the Witwatersrand, who is Professor Peter Cleaton-Jones at telephone number: (011) 717-2301

## Annex E: Information sheet for group interview with community health workers

**Formal Title:** Implementing comprehensive, integrated, community-based health care for under-served, vulnerable communities in South Africa: A practical, evidence-informed model.

Hello, and thank you for your time today.

My name is ________. I work for the Centre for Health Policy at the University of the Witwatersrand. I am a researcher in the Batlhokomedi project, and I would like to talk to you today as part of this project.

What is this research about?

We are conducting this research to understand how community health worker / WBOT programmes operate. We know that there are some innovative WBOT programmes in Sedibeng and we are keen to learn more about your programmes here. I know you are community health workers, so we would like to hear about your experiences of being a CHW, both the successes and challenges.

The group interview should take about 1 hour.



For peer review only - http://bmjopen.bmj.com/site/about/guidelines.xhtml

For peer review only

V0.95; 12 September 2016

Voluntary Participation

It is up to you to decide whether you want participate in this study. If you agree to help with this research, and later change your mind, you are free to leave the group interview at any time, without any consequences.

Risks

There are no risks associated with talking to me today.

Confidentiality

No one other than my team members will be allowed to see the record of the discussion. We will not use your name in any reports of this work. My notes and audio recordings will be kept on a secure university computer, and will be destroyed once our research is complete. We (the researchers) will keep the discussion confidential, and we would like to ask all the participants to do the same. However, as researchers we cannot guarantee this.

Approval for and benefits of this work

The study has been approved by the ethics committee of University of the Witwatersrand, and Gauteng Province. We hope the study will contribute new ideas on how to improve community health worker programmes.

If you agree to participate in the group interview, we would also like your permission to record the interview as it really helps later in the office.

<u>What if I have any questions?</u>

You are free to ask me any question about this research. If you have any further questions about the study, you are free to contact Julia De Kadt on Julia.DeKadt2@wits.ac.za, or 011-717-3434 or 074 336 9411.

If you are concerned about anything to do with the study in general, or wish to make a complaint, you can contact the chair of the ethics committee at the University of the Witwatersrand, who is Professor Peter Cleaton-Jones at telephone number: (011) 717-2301

## Tool E: Group interview with Community Health Worker team

This will usually take place during the working day of the Community Health Worker's working day after they have had refreshments provided by the research team. The venue will usually be a quiet room in the clinic or health post. The supervising nurse will not be present.

The facilitator and the scribe conducting the group interview will introduce themselves. The facilitator will facilitate the discussion and the scriber will take field notes that will complement the audio-recording (e.g. behaviours, interruptions). The facilitator will encourage discussion between the Community Health Workers about what works well or not and why.



For peer review only - http://bmjopen.bmj.com/site/about/guidelines.xhtml

V0.95; 12 September 2016

**At the start of the session ask the participants to complete the questionnaire**

*Check questionnaires are complete (facilitator and scribe to provide assistance where necessary). Check with group that they are ready and turn on the audio recorder announcing that you are doing this.*

Introduction

**Thank you again for agreeing to be interviewed. To start the session I am going to ask each of you in turn to tell me how you came to join the team?**

*Go around every participant in turn*

**Thank you. I am now going to ask you about your work with WBOT. Please all help to answer the questions.**

**I would like you to tell me about what you do day to day.**

Please tell me about how a typical day at work starts, what do you do?
What happens during a typical day?
What happens at the end of the day?

**Do you plan your work? How?**

How do you plan your work or decide which households you'll visit on a particular day? (do you do this with your colleague(s) who pairs up with you?)
How do you keep track of the households where you need to follow up on a patient?

Do you do different tasks on different days of the week? What are these?

**Can you please tell us a bit more about household registration?**

When do you do household registration?

How often do you do household registration? How long does it take?

Is there any form you use when doing household registration?

What questions do you ask? What do you focus on?

Is there more than one kind of household registration?

What happens to these forms afterwards? Do you use them later on?

What happens to the information you collect during household registration? (probe, are there new patients identified and assigned after household registration? How are they assigned?)

**Can you please tell us a bit more about home based care work?**

Do you do any home based care? When does it happen?

How much of your time do you spend doing home based care work?


For peer review only - http://bmjopen.bmj.com/site/about/guidelines.xhtml

**Please can you describe for us the data reporting system here?**

What information do you record when you are out in the community?
What do you do with this information, and when?
How do you prepare your statistics?

What challenges do you experience with preparing your statistics?

**Thank you. Now please tell me about your pack.**
What is in it?
When do you use the contents? What for?
How are the consumables (name them e.g. forms, dressings) replenished?
How is the equipment (name it e.g. glucometer, bp machine,) maintained in working order?

**What other support do you get to make your work easier?**

Material resources, e.g. airtime, a meeting place, a place to store your files? Are these sufficient? If not, please explain?

Support from colleagues, your team leader, the clinic staff, from the community?

**What do you do when you need help?**
What type of situations do you find when you need help?
What help is available?
How do you access the help?
Is the help you receive sufficient? Please explain.

**What happens when a team member is struggling or can't visit all of their households, for example because of sickness?**
How does the team work when one member can't do their job?

**What achievements in your work are you proud of?**
What do you think has enabled you to achieve this?
What would enable you to achieve more?

**What are the challenges that you face?**
What dilemmas do you face in your day to day work?
Please tell me about times when you have faced conflicting demands?
Possible probes:
- Workload
- Completing paper work
- Preparing statistics
- Payment, Clocking system
- Transport
- Appreciation, recognition
- Conflict with community
- Conflict with OTL or facility/health post staff
- Working after hours & over weekend (types of tasks; how often, how long)

**Job security**


For peer review only - http://bmjopen.bmj.com/site/about/guidelines.xhtml

Do you feel that your job is secure? Have you encountered or heard problems about contract renewal with SmartPurse?

What do you feel about your prospects for career development and promotions?

Are you worried that you might face job loss or layoff?

If you were laid off, are you worried that you would have difficulty finding a suitable job?

How do you feel about SmartPurse?

**Please could you tell us a bit about the strike?**

What happened?
Have the issues been resolved?

**How do you relate to the community?**

What does the community think of you as a team?
How does the community share complaints, concerns or compliments about the WBOT team with you?
Are there other organisations providing health care in the community and what do they do?
How do you relate to them?

**For CHWs who were previously working for an NGO:**
**Has your work changed since you moved from the NGO to the WBOT team? If so, how?**

Which people in the community were you looking after when you worked for the NGO?

What kind of tasks did you do when you worked for the NGO?

Who was supervising you when you worked for the NGO?

What resources did you have when you worked for the NGO?

What challenges did you experience when you worked for the NGO?

How is your work different now that you work in the WBOT team?

For peer review only

**<u>Ending the focus group</u>**

Thank you. I think we've covered all of the questions that I wanted to ask you today. Do you have anything else you wanted to add?

**<u>Topics we would like to hear about from the interviewees:</u>**

guidelines, policies and protocols;
registration of households, case finding;
home visit records, numbers, planning;
recent health campaigns and role of Community Health Workers;
access to supplies, transport, advice, equipment, space to meet and store;
referral management and feedback;
language barriers;
safety and security of CHW in the community;
previous training and experience of CHW;
current/recent in role training
payment ;



For peer review only - http://bmjopen.bmj.com/site/about/guidelines.xhtml

supervision from team leader;
households refusing entry;
liaising with clinic staff;
communication with other organisations providing care in the community.

**Suggested generic prompts to use to encourage participants to continue:**

*To get going on a topic if they are unsure of where to start or what to say:*
Start wherever you want
Start with what you think is most important
Why not start by telling me what happens in a day, starting at the beginning

*To go into more depth or to encourage continuation of their account*
Please tell me more about that
Please explain that for me
Why was that?
What did you feel about that?
What happened then?
Please can you tell me more about (person, role, place, organisation, activity etc.)

**CHW Focus Group Discussion Questionnaire** **FGD ID:** ________________________

| | | |
|---|---|---|
| 1 | Date of interview | |
| 2 | Name of Health post / clinic | |
| 3 | Age (Years) | |
| 4 | Gender (please circle) | Male Female |
| 5 | How many years have you worked as a CHW? | |
| 6 | How many years have you worked as a CHW in this area? | |
| 7 | How many years have you been a part of this team? | |
| 8 | How many households are you responsible for ? | |

**Travelling from home to the clinic or health post**

| | | |
|---|---|---|
| 9 | How long does it take you to get from your house to the clinic or health post where you are based? | ______ hours _______ minutes |
| 10 | How do you mainly travel from your home to the clinic or health | Walk<br>Taxi |



For peer review only - http://bmjopen.bmj.com/site/about/guidelines.xhtml

| | | |
|---|---|---|
| | post where you are based? Please circle, and describe if other | Other: ________________ |
| 11 | If you travel from your home to the households you visit by taxi, please provide the fare, in Rands. | |

**Travel from your home to the households you care for**

| | | |
|---|---|---|
| 12 | If you travelled directly from your home to a household you care for, on average how long would this take you? | ______ hours _______ minutes |
| 13 | If you needed to travel directly from your home to a household you car for, what mode of transport would you mainly use? | Walk<br>Taxi<br>Other: ________________ |
| 14 | If you would travel from your home to a household you care for by taxi, please provide the fare, in Rands. | |

**Education, training and other employment**

| | | | |
|---|---|---|---|
| 15 | What is your highest level of education (please circle) | Some primary school<br>Completed primary school<br>Some secondary school<br>Completed secondary school (passed matric)<br>Some tertiary education | |
| 16 | If you have completed some tertiary education, please describe | | |
| 17 | Please describe any CHW training you have done | | |
| 18 | Do you have any other job apart from this one? (Please circle) | Yes | No |
| 19 | If you do have another job, please describe this | | |

For peer review only

V0.95; 12 September 2016

## Annex G: Information Sheet for interviewing referred householder

**Formal Title:** Implementing comprehensive, integrated, community-based health care for under-served, vulnerable communities in South Africa: A practical, evidence-informed model.

Hello, and thank you for your time today.

My name is ________. I work for the Centre for Health Policy at the University of the Witwatersrand. I am a researcher in the Batlhokomedi project, and visited your household about a month ago with a community health worker. I am here today just to follow up with you after that visit.

<u>What is this research about?</u>
We are conducting this research to understand how community health worker / WBOT programmes operate. We are here to learn more about the community health workers in Sedibeng, by talking to people like yourself about what the CHWs do.

The interview should take about 40 mins.

Voluntary Participation

It is up to you to decide whether you want to participate in this study. If you agree to help with this research, and later change your mind, you are free to end the interview at any time, without any consequences.

Risks

There are no risks associated with talking to me today.

Confidentiality

No one other than my team members will be allowed to see the record of the interview. No contents of the interview will be shared with the clinic or health post, or any Community Health Workers, including the Community Health Worker who brought me here. We will not use your name in any reports of this work. We will use a code instead of your name e.g. A or B. My notes and audio recordings will be kept on a secure university computer server. Participant codes will be kept in a locked filing cabinet under the care of the project site manager, and will be destroyed once our research is complete.

Approval for and benefits of this work

For peer review only

V0.95; 12 September 2016

The study has been approved by the ethics committee of University of the Witwatersrand, and Gauteng Province. We hope the study will contribute new ideas and insights regarding the provision of community health worker services.

If you agree to be interviewed, we would also like your permission to record the interview as it really helps later in the office.

<u>What if I have any questions?</u>

You are free to ask me any question about this research. If you have any further questions about the study, you are free to contact Julia De Kadt on Julia.DeKadt2@wits.ac.za, or 011-717-3434 or 074 336 9411.

If you are concerned about anything to do with the study in general, or wish to make a complaint, you can contact the chair of the ethics committee at the University of the Witwatersrand, who is Professor Peter Cleaton-Jones at telephone number: (011) 717-2301

## Tool G: Interviews with referred householder

Introduction

Thank you again for agreeing to be interviewed. Are you happy to start?

**First, could you please tell me a little bit about yourself?**
Probes:
Age
Gender
Language spoken
Number of people in household
Are you working?
If yes, what type of work do you do?
What other sources of income are there in the household (child support grants, pension, disability)?

**Thank you. Now, let's talk a little bit about the Community Health Worker's visit. Please tell me about what happened when the Community Health Worker came to your house.**
Probes:
What happened?
Can you describe how you felt about the visit that day?
Were any other household members around?
If so, what did other household members say or do?
What did the CHW suggest you do (in terms of your health/the issue you were experiencing)?
What did the CHW say she would do (in terms of your health/the issue you were experiencing)?

*If the CHW suggested the patient should do something:*
**Were you able to do what the CHW suggested you do?**
Probes:
What did you actually do?


For peer review only - http://bmjopen.bmj.com/site/about/guidelines.xhtml

V0.95; 12 September 2016

Why?
What made it easy or difficult to do what the Community Health Worker suggested?
How helpful did you find the Community Health Worker's suggestion?

**For householders who have not taken action based on the Community Health Worker recommendation:**
Why didn't you do what the community health worker suggested?

*If the CHW said she would do something:*
**Did the CHW do what she said she would do?**
If the CHW didn't do what she said she would, do you know why? Do you think she will still do what she said?
Did the CHW do anything else?
Has the CHW made any other visits/follow-ups after the visit we've been discussing?
If so, when, and what did she do during those visits?

**Could you please tell me about how you are doing now (with regards to your health/the issue you were experiencing)?**
Probes (use only the appropriate ones):
What health care are you receiving now for your health?
Follow up visits, treatments, tests
What services are you receiving related to the issue you were experiencing?
**Could we talk a bit about the CHW's service more generally?**
Probes:
Is their service useful?
Can you give me any other examples of when they've assisted you?
Has it changed (over time)? How?
How could it be improved?
When did they start coming to see you?
How often do they usually come to see you?

<u>**Ending the interview**</u>
Thank you. I think we've covered all of the questions that I wanted to ask you today. Do you have anything else you wanted to add?

**Suggested generic prompts to use to encourage participants to continue:**

*To get going on a topic if they are unsure of where to start or what to say:*
Start wherever you want
Start with what you think is most important
Why not start by telling me what happens in a day, starting at the beginning

*To go into more depth or to encourage continuation of their account*
Please tell me more about that
Please explain that for me
Why was that?
What did you feel about that?
What happened then?
Please can you tell me more about (person, role, place, organisation, activity etc.)


For peer review only - http://bmjopen.bmj.com/site/about/guidelines.xhtml
For peer review only

## COREQ (COnsolidated criteria for REporting Qualitative research) Checklist

A checklist of items that should be included in reports of qualitative research. You must report the page number in your manuscript where you consider each of the items listed in this checklist. If you have not included this information, either revise your manuscript accordingly before submitting or note N/A.

| Topic | Item No. | Guide Questions/Description | Reported on Page No. |
|---|---|---|---|
| **Domain 1: Research team and reflexivity** | | | |
| *Personal characteristics* | | | |
| Interviewer/facilitator | 1 | Which author/s conducted the interview or focus group? | 5 |
| Credentials | 2 | What were the researcher's credentials? E.g. PhD, MD | 6 |
| Occupation | 3 | What was their occupation at the time of the study? | 6 |
| Gender | 4 | Was the researcher male or female? | 6 |
| Experience and training | 5 | What experience or training did the researcher have? | 6 |
| *Relationship with participants* | | | |
| Relationship established | 6 | Was a relationship established prior to study commencement? | 6 |
| Participant knowledge of the interviewer | 7 | What did the participants know about the researcher? e.g. personal goals, reasons for doing the research | 5 |
| Interviewer characteristics | 8 | What characteristics were reported about the inter viewer/facilitator? e.g. Bias, assumptions, reasons and interests in the research topic | 5 |
| **Domain 2: Study design** | | | |
| *Theoretical framework* | | | |
| Methodological orientation and Theory | 9 | What methodological orientation was stated to underpin the study? e.g. grounded theory, discourse analysis, ethnography, phenomenology, content analysis | 6 |
| *Participant selection* | | | |
| Sampling | 10 | How were participants selected? e.g. purposive, convenience, consecutive, snowball | 6 |
| Method of approach | 11 | How were participants approached? e.g. face-to-face, telephone, mail, email | 6 |
| Sample size | 12 | How many participants were in the study? | 6 |
| Non-participation | 13 | How many people refused to participate or dropped out? Reasons? | 6 |
| *Setting* | | | |
| Setting of data collection | 14 | Where was the data collected? e.g. home, clinic, workplace | 5 |
| Presence of non-participants | 15 | Was anyone else present besides the participants and researchers? | 6 |
| Description of sample | 16 | What are the important characteristics of the sample? e.g. demographic data, date | 5 |
| *Data collection* | | | |
| Interview guide | 17 | Were questions, prompts, guides provided by the authors? Was it pilot tested? | 6 |
| Repeat interviews | 18 | Were repeat inter views carried out? If yes, how many? | 5 |
| Audio/visual recording | 19 | Did the research use audio or visual recording to collect the data? | 6 |
| Field notes | 20 | Were field notes made during and/or after the inter view or focus group? | 6 |
| Duration | 21 | What was the duration of the inter views or focus group? | 5 |
| Data saturation | 22 | Was data saturation discussed? | 6 |
| Transcripts returned | 23 | Were transcripts returned to participants for comment and/or | 7 |

For peer review only

| Topic | Item No. | Guide Questions/Description | Reported on Page No. |
|---|---|---|---|
| | | correction? | |
| **Domain 3: analysis and findings** | | | |
| *Data analysis* | | | |
| Number of data coders | 24 | How many data coders coded the data? | 6 |
| Description of the coding tree | 25 | Did authors provide a description of the coding tree? | 6 |
| Derivation of themes | 26 | Were themes identified in advance or derived from the data? | 6 |
| Software | 27 | What software, if applicable, was used to manage the data? | 6 |
| Participant checking | 28 | Did participants provide feedback on the findings? | 7 |
| *Reporting* | | | |
| Quotations presented | 29 | Were participant quotations presented to illustrate the themes/findings? Was each quotation identified? e.g. participant number | 7-12 |
| Data and findings consistent | 30 | Was there consistency between the data presented and the findings? | 7-12 |
| Clarity of major themes | 31 | Were major themes clearly presented in the findings? | 7-12 |
| Clarity of minor themes | 32 | Is there a description of diverse cases or discussion of minor themes? | 12 |

Developed from: Tong A, Sainsbury P, Craig J. Consolidated criteria for reporting qualitative research (COREQ): a 32-item checklist for interviews and focus groups. *International Journal for Quality in Health Care*. 2007. Volume 19, Number 6: pp. 349 – 357

**Once you have completed this checklist, please save a copy and upload it as part of your submission. DO NOT include this checklist as part of the main manuscript document. It must be uploaded as a separate file.**

For peer review only
